# Globally prevalent PfMDR1 mutations modulate *Plasmodium falciparum* susceptibility to artemisinin-based combination therapies

**DOI:** 10.1038/ncomms11553

**Published:** 2016-05-18

**Authors:** M. Isabel Veiga, Satish K. Dhingra, Philipp P. Henrich, Judith Straimer, Nina Gnädig, Anne-Catrin Uhlemann, Rowena E. Martin, Adele M. Lehane, David A. Fidock

**Affiliations:** 1 Department of Microbiology and Immunology, Columbia University Medical Center, Room 1502 HHSC, 701 West 168th Street, New York, New York 10032, USA; 2 Life and Health Sciences Research Institute (ICVS), School of Health Sciences and ICVS/3B's—PT Government Associate Laboratory, University of Minho, Gualtar Campus, 4710-057 Braga, Portugal; 3 Division of Infectious Diseases, Department of Medicine, Columbia University Medical Center, Box 82, 630 West 168th Street, New York, New York 10032, USA; 4 Research School of Biology, Linnaeus Way, The Australian National University, Acton Australian Capital Territory 2601, Australia

## Abstract

Antimalarial chemotherapy, globally reliant on artemisinin-based combination therapies (ACTs), is threatened by the spread of drug resistance in *Plasmodium falciparum* parasites. Here we use zinc-finger nucleases to genetically modify the multidrug resistance-1 transporter PfMDR1 at amino acids 86 and 184, and demonstrate that the widely prevalent N86Y mutation augments resistance to the ACT partner drug amodiaquine and the former first-line agent chloroquine. In contrast, N86Y increases parasite susceptibility to the partner drugs lumefantrine and mefloquine, and the active artemisinin metabolite dihydroartemisinin. The PfMDR1 N86 plus Y184F isoform moderately reduces piperaquine potency in strains expressing an Asian/African variant of the chloroquine resistance transporter PfCRT. Mutations in both digestive vacuole-resident transporters are thought to differentially regulate ACT drug interactions with host haem, a product of parasite-mediated haemoglobin degradation. Global mapping of these mutations illustrates where the different ACTs could be selectively deployed to optimize treatment based on regional differences in PfMDR1 haplotypes.

## Abstract (web summary)

Antimalarial chemotherapy relies on combination therapies (ACTs) consisting of an artemisinin derivative and a partner drug. Here, the authors study the effects of globally prevalent mutations in a multidrug resistance transporter (PfMDR1) on the parasite's susceptibility to ACT drugs.

## Graphical abstract

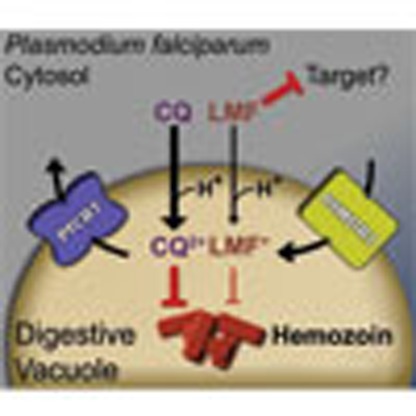

Malaria in 2015 was responsible for an estimated 214 million cases and 438,000 deaths^1^. Fatal cases, resulting primarily from infection with the Apicomplexan parasite *Plasmodium falciparum*, are half those occurring 15 years ago, primarily as a result of the global adoption of highly effective artemisinin (ART)-based combination therapies (ACTs). These ACTs combine a fast acting but rapidly cleared ART derivative with a longer-lasting partner drug. The major partner drugs are lumefantrine (LMF, which combined with artemether (ATM) constitutes the most widely used ACT), mefloquine (MFQ, paired with artesunate (AS)), amodiaquine (ADQ, also paired with AS) and piperaquine (PPQ, combined with the active ART metabolite dihydroartemisinin (DHA)). These drugs share certain chemical features such as the 4-aminoquinoline ring present in ADQ and PPQ (as well as chloroquine, CQ) or the arylaminoalcohol group present in LMF and MFQ (Fig. 1), and appear to interact with the parasite haem detoxification pathway^2^. These interactions might only be a part of complex modes of action, such as for MFQ (and possibly LMF) where activity against a cytosolic target (or targets) has been evoked^3^. In the case of ART derivatives, these are activated by the central Fe^2+^ atom present in haem on its release from haemoglobin^4^.

In a direct threat to malaria control programmes, ACT resistance is now emerging, led by *P. falciparum* resistance to the ART derivatives. This resistance, which manifests as relatively slow rates of parasite clearance following treatment, is now widespread in the Greater Mekong Sub-region^5^, raising concerns about its possible spread into the African continent where malaria exerts its heaviest toll. Epidemiological and molecular genetic studies have recently shown that ART resistance is primarily mediated by mutations in the propeller domain of the *P. falciparum* K13 kelch protein^5^,^6^,^7^,^8^. Reduced ART efficacy in turn places increased selective pressure on the ACT partner drugs, placing them at greater risk of failing. Indeed, PPQ resistance has now emerged in *K13*-mutant parasites, resulting in treatment failure with DHA+PPQ—the first-line drug in Cambodia and several neighbouring countries^9^,^10^,^11^. Despite an active antimalarial drug discovery and development pipeline, there is at present no drug ready to take the place of ACTs should they begin to fail globally^12^.

Delineating the role of the *P. falciparum* multidrug resistance-1 gene *pfmdr1* is of particular relevance due to its suspected involvement in parasite susceptibility to each of the ACT partner drugs mentioned above, as well as its association with altered susceptibilities of trophozoite-stage parasites to ART derivatives^13^,^14^,^15^. These findings, acquired in molecular epidemiology studies, implicate mutant PfMDR1 in multidrug resistance phenotypes. The interpretation of these earlier studies, which largely relied on typing single nucleotide polymorphisms (SNPs), is tempered by the lack of complete PfMDR1 haplotypes. Studies of isogenic parasites engineered to differ only at their *pfmdr1* locus have the benefit of reducing the genetic complexity and attributing changes in drug susceptibility to the introduced sequence changes. Such studies have become more technically feasible since the advent of genome editing in *P. falciparum*, which significantly increases the speed and precision with which SNPs can be exchanged at a gene of interest^16^.

PfMDR1 is an ATP-binding cassette transporter and a homologue of the human multidrug-resistance-conferring P-glycoprotein. It is 1,419 amino acids in length (for the 3D7 isoform) and contains 12 putative transmembrane domains. PfMDR1 and the pleiotropic drug resistance transporter PfCRT both reside on the membrane of the parasite's digestive vacuole (DV), where they are thought to regulate the flux of solutes across this membrane^17^. Their functional relatedness is evidenced by the linkage disequilibrium seen between certain combinations of *pfmdr1* and *pfcrt* alleles^18^,^19^. This might serve to maximize drug resistance phenotypes and/or reflect compensatory mutations that reduce any negative impact of mutations in one transporter on DV physiology or parasite growth. In a genetic cross between clones of South American (HB3, CQ sensitive) and Asian (Dd2, CQ resistant) origin, *pfcrt* was identified as the primary determinant of CQ resistance, with the K76T mutation being ubiquitous to the CQ-resistant progeny^20^. A separate cross between CQ-resistant clones from South America (7G8) and Africa (GB4; having a higher degree of CQ resistance than 7G8) revealed that the South American *pfcrt* and *pfmdr1* alleles combine to confer high-level resistance to monodesethyl-ADQ (md-ADQ), the active ADQ metabolite^19^.

Studies of *pfmdr1* have identified five globally prevalent amino acid mutations. The amino-terminal mutations (N86Y and Y184F) are more common to Asian and African parasites, whereas the three carboxy-terminal mutations (S1034C, N1042D and D1246Y) are found more often in South American isolates (D1246Y is nonetheless present in ∼3% of the 1,502 African genomes recently sequenced by the MalariaGEN consortium; see below). The ability of PfMDR1 variants to influence antimalarial drug potency is supported by heterologous expression systems that provide evidence of drug transport by certain PfMDR1 isoforms^21^,^22^.

Earlier transfection studies have delineated the role of the C-terminal PfMDR1 mutations in modulating *P. falciparum* response to antimalarial drugs including MFQ, ART, CQ and quinine (QN)^23^,^24^. Attempts to modify the N-terminal mutations were unsuccessful, presumably because the former single-site, cross-over-based strategies necessitated changes to the *pfmdr1* regulatory elements that proved unsuitable for parasite growth^24^. That restriction has been negated with the development of zinc-finger nucleases (ZFNs), which permit precise gene editing by triggering a specific double-stranded break adjacent to the targeted SNP. Homology-directed recombination can then be leveraged to repair the DNA lesion, without requiring the modification of any gene regulatory elements or the permanent integration of a selectable marker^25^. This approach has been successfully used to define the role of the resistance mediators *pfcrt*, *k13* and *pi4k*^7^,^26^,^27^,^28^. Here we report its successful use to engineer *pfmdr1* N86Y and Y184F mutations in parasite strains that express the two major CQ resistance-conferring PfCRT variants. We also use publicly available data from 2,512 *P. falciparum* genomes to explore the distribution of PfMDR1 haplotypes at positions 86 and 184 in endemic regions. Our results show that the N86Y mutation contributes to resistance to CQ and ADQ, while sensitizing parasites to LMF, MFQ and DHA. In contrast, the Y184F mutation has a limited impact. When combined with the genome analyses, these findings help inform the selection of optimal treatment regimens based on an assessment of local drug selective pressures and the geographic distribution of PfMDR1 haplotypes.

## Results

### Geographical distribution of PfMDR1 haplotypes

**Figure 2:**
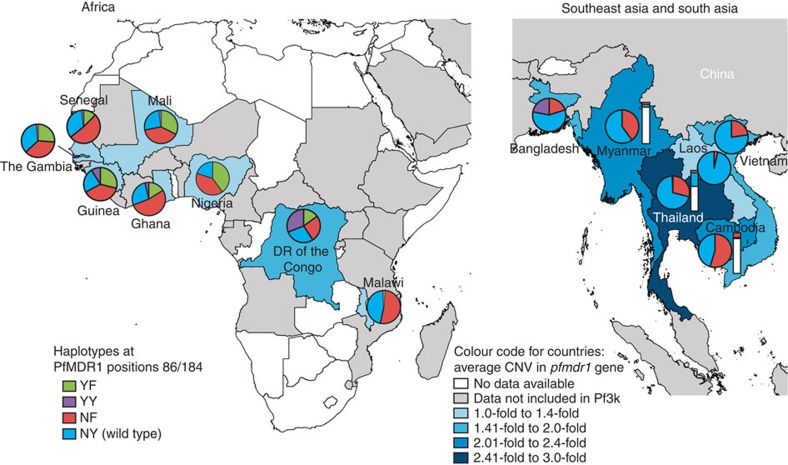
Geographical distribution of PfMDR1 haplotypes at residues 86 and 184 and *pfmdr1* copy number variations. Data were compiled using the Sanger Pf3k data set (www.malariagen.net/apps/pf3k/release_3/index.html)^8^,^29^ and comprised 2,512 genomes. Pie charts represent proportions of PfMDR1 haplotypes (numerical data per country in Supplementary Table 1). Country colourings show average *pfmdr1* amplification compared with the 3D7 reference genome with single-copy *pfmdr1*. Vertical bars in Southeast Asia show the proportions of haplotypes represented among *pfmdr1* copy number variants. Vertical bars colouring: White: no *pfmdr1* amplification; blue: *pfmdr1* amplification plus NY haplotype; red: *pfmdr1* amplification plus NF haplotype. Countries where PfMDR1 individual polymorphism data are available via WWARN (www.wwarn.org/tracking-resistance/molecular-surveyor-pfmdr1-pfcrt) are indicated in grey (data not evaluated herein due to the lack of combined N86 and Y184 status in the full data set).

Recent advances in whole-genome sequencing and genome analysis, applied to thousands of *P. falciparum* genomes by the MalariaGEN consortium^8^,^29^, permit a detailed investigation of PfMDR1 haplotypes across malaria-endemic regions of Southeast Asia and Africa (large-scale genome data from South American and Western Pacific strains are not yet available). By analysing 2,512 parasite genomes made available by this consortium (www.malariagen.net/pf3k), we determined the frequencies of the four PfMDR1 haplotypes differing at residues 86 and 184 across multiple countries (Fig. 2 and Supplementary Table 1). We used the N86/Y184 (NY) haplotype of the drug-sensitive reference strain 3D7 as the wild-type comparator. Results show three of the four haplotypes at positions 86 and 184 being relatively common across West Africa, with only N86Y/Y184 PfMDR1 (YY) being relatively rare. All four were observed in the Congo in Central Africa, while Malawi in East Africa essentially lacked the N86Y mutation and harboured only N86/Y184F (NF) and NY. In Southeast Asia the N86Y mutation was also strikingly absent (although it remained to the west in Bangladesh) and the Y184F mutation varied in prevalence from >50% in Cambodia to <4% in Laos. Southeast Asian isolates quite often also harboured multiple copies of *pfmdr1* (155/1,010 genomes), more so in parasites with the NY haplotype and never with the N86Y mutation (Fig. 2 and Supplementary Table 1). This was most apparent in Thailand, where nearly 40% of all genomes had amplified *pfmdr1*, with as many as five tandem copies. Amplified *pfmdr1* was exceedingly rare in Africa (observed in 12/1,502 genomes), potentially because of a fitness cost that is not conducive to its maintenance in African high-endemicity settings^30^. Parallel assessment of *pfcrt* codon 76 revealed that 98% of all sampled parasites in Southeast Asia carried the K76T mutation (Table 1 and Supplementary Table 1). Surveys of African parasites revealed far greater heterogeneity. Parasites from Malawi were exclusively K76, consistent with strict removal of CQ pressure^31^, whereas other countries such as the Congo maintained the K76T CQ resistance marker in 71% of the sampled isolates.

### Targeted modification of *pfmdr1*

Using customized ZFNs, we generated two sets of *pfmdr1*-edited parasites at codons 86 and 184 (Table 2). These sets represented the two major geographic variants of mutant PfCRT, namely SVMNT and CVIET (spanning codons 72–76). ZFNs were engineered to bind neighbouring sites on opposite strands of *pfmdr1*, producing a double-stranded break 42 bp downstream of the start codon. As previously documented, double-stranded break repairs proceed via homology-directed recombination in *P. falciparum*, which lacks the alternative non-homologous end-joining repair pathway^25^. Our homology-driven repair template consisted of a 2.4-kb *pfmdr1* fragment that encompassed codons 86 and 184. To prevent ZFNs from cleaving the plasmid or the edited locus, three silent (non-synonymous) mutations were engineered into the donor sequence at the ZFN-binding site.

**Figure 3:**
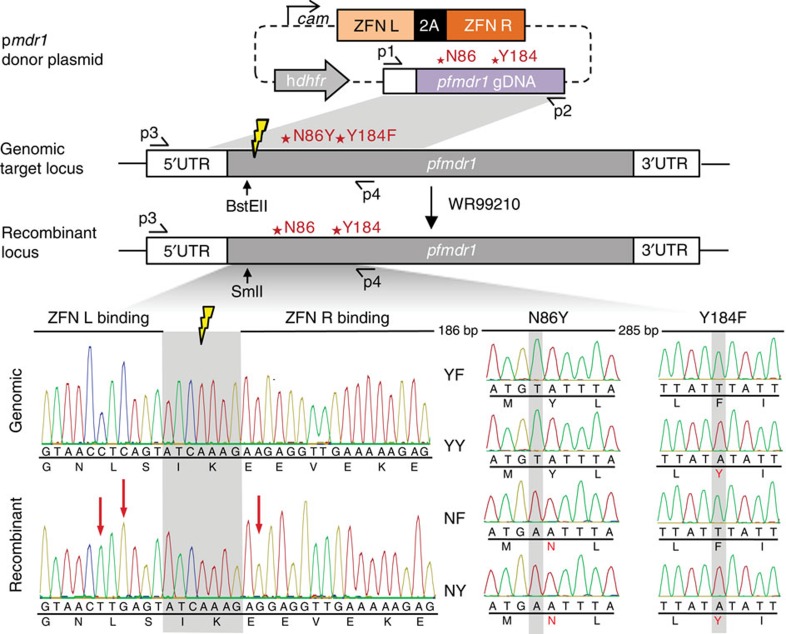
Schematic of the ZFN-based *pfmdr1* editing strategy. The p*mdr1* plasmid expresses the 2A-linked *pfmdr1*-specific ZFN pair from the calmodulin promoter and the human *dhfr* selectable marker. These ZFNs create a double-stranded break that can be repaired using the plasmid-borne homologous donor sequence that extends 0.6 kb upstream and 1.8 kb downstream of the target site (yellow thunderbolt). The four p*mdr1* transfection plasmids carry three silent mutations at the binding sites to prevent ZFNs cleaving the plasmids or the edited sequence. These plasmids encode the four haplotypes at residues 86 and 184. Shown is the example of transfecting a N86/Y184 parasite with the p*mdr1*^NY^ plasmid (Table 2). Chromatograms show sequence analysis of genomic and recombinant DNA samples. Mutations at the ZFN binding site (red arrows) and the N86Y and Y184F change of codons are indicated.

*P. falciparum*-infected red blood cells (RBCs), predominantly ring stages, were electroporated with the p*mdr1* ZFN plasmids that also express human dihydrofolate reductase (hDHFR). This selectable marker mediates resistance to WR99210, which was applied for 4–6 days, to select for transformed parasites. PCR screening was used to identify edited parasites, which were cloned by limiting dilution. Figure 3 illustrates the ZFN strategy and shows representative electropherograms of *pfmdr1* editing events.

Editing of *pfmdr1* was performed with the *P. falciparum* NF10 and KC5 clones. These are progeny of the genetic cross between 7G8 (Brazil) and GB4 (Ghana) parasites (Table 2)^32^. Both progeny lines inherited a single copy of the GB4 *pfmdr1* allele, which encodes the N86Y/Y184F haplotype (YF). NF10 inherited the CVIET PfCRT haplotype that is predominant in Asia and Africa, whereas KC5 harbours the SVMNT haplotype that is highly prevalent in South America and the Western Pacific. These progeny differ in their level of CQ resistance and their response to QN and ADQ^19^ (see below). Parasites were transfected with four different p*mdr1* plasmids to generate the four different haplotype combinations, namely YF, YY, NF and NY, in both *pfcrt* genetic backgrounds. The resulting lines are denoted NF10^*mdr1*-YF^ (recombinant control), NF10^*mdr1*-YY^, NF10^*mdr1*-NF^, NF10^*mdr1*-NY^, KC5^*mdr1*-YF^ (recombinant control), KC5^*mdr1*-YY^, KC5^*mdr1*-NF^ and KC5^*mdr1*-NY^ (Table 2). Quantitative PCR analysis revealed that all parasites continued to express a single copy of *pfmdr1*, with no change introduced during gene editing (Supplementary Table 2).

*In vitro* drug susceptibility assays were performed on the different *pfmdr1* recombinant lines. Assays were performed with two independent clones for each line, with the exception of the recombinant control lines (NF10^*mdr1*-YF-1^ and KC5^*mdr1*-YF-1^) and the KC5^*mdr1*-YY^ line for which only single clones were recovered. Drug susceptibility assays used a range of ten compound concentrations diluted twofold and were performed on five to ten separate occasions in duplicate. Numbers of surviving parasites were quantified using flow cytometry. IC_50_ and IC_90_ values were determined for LMF, MFQ, DHA, PPQ, CQ, monodesethyl-CQ (md-CQ; the major *in vivo* metabolite of CQ), md-ADQ and QN.

**Figure 4:**
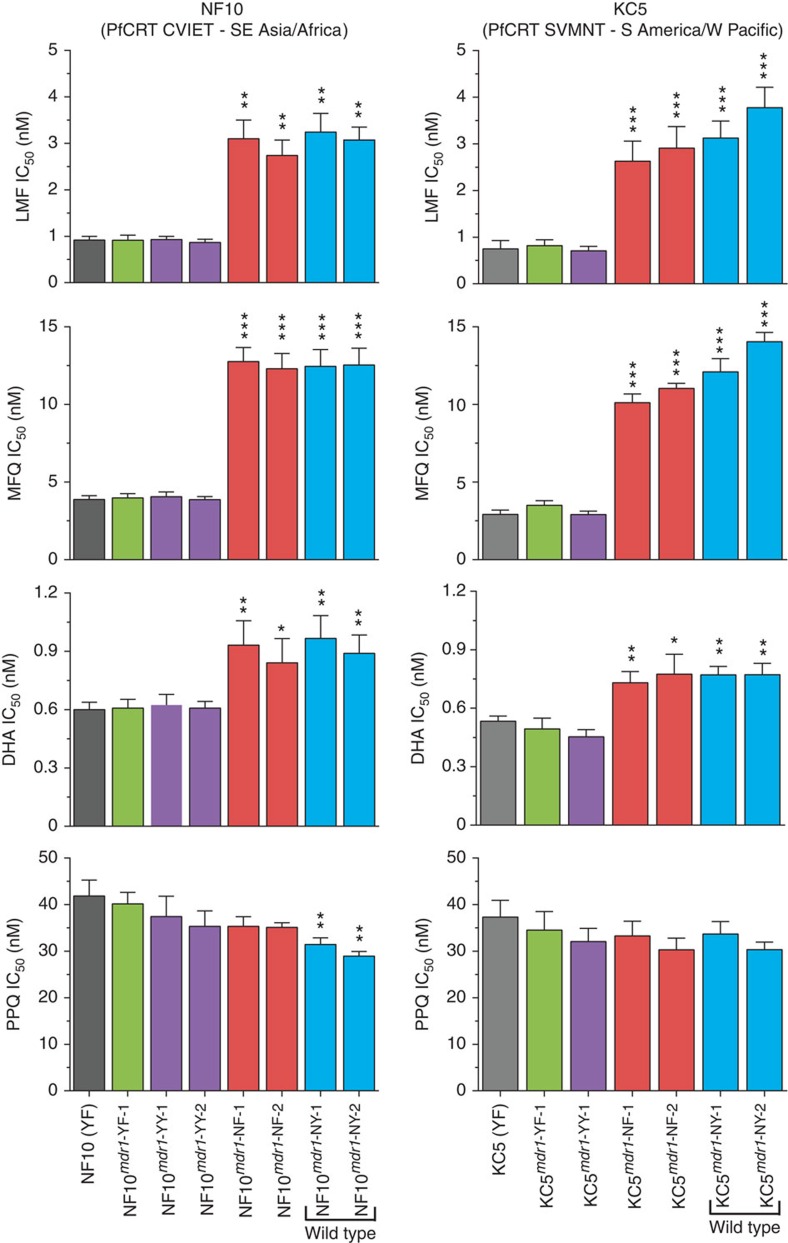
*In vitro* IC_50_ response of *pfmdr1*-modified and parental lines. IC_50_ values (nM) were determined by incubating parasites for 72 h across a range of drug concentrations. Parasite growth was determined by measuring parasitaemia using flow cytometry with cells stained with Mito Tracker Deep Red and SYBR Green. Mean±s.e.m. IC_50_ values are presented for lumefantrine (LMF), mefloquine (MFQ), dihydroartemisinin (DHA) and piperaquine (PPQ). Five to ten assays were performed for each drug (Supplementary Tables 3 and 4). Statistical evaluations comparing mutant *pfmdr1*-modified lines against recombinant control lines of the same genetic backgrounds (NF10^*mdr1*-YF-1^ and KC5^*mdr1*-YF-1^ for NF10 and KC5 lines, respectively) were performed using two-tailed Mann–Whitney *U*-tests. *P<0.05, **P<0.01 and ***P<0.001.

**Figure 5:**
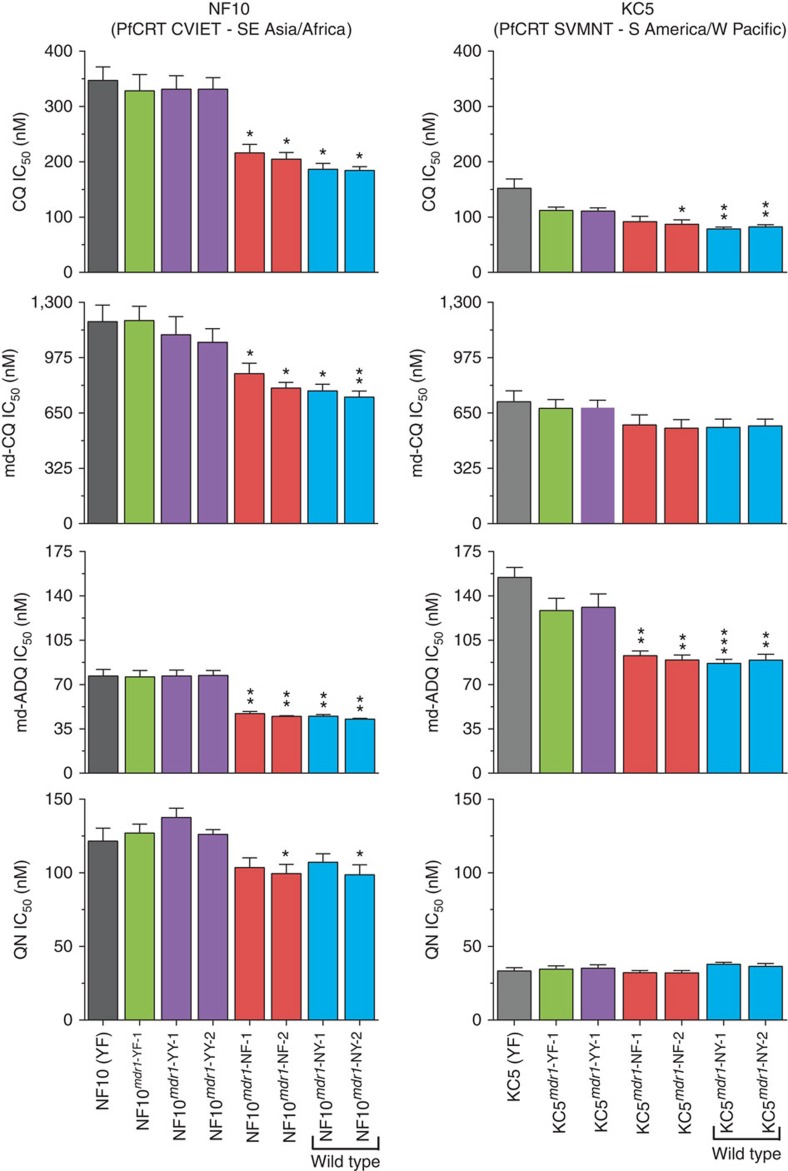
*In vitro* IC_50_ response of *pfmdr1*-modified and parental lines. IC_50_ values (nM) are presented for CQ, md-CQ, md-ADQ and QN. Five to ten assays were performed for each drug (Supplementary Tables 3 and 4). Statistical evaluations comparing mutant *pfmdr1*-modified lines against recombinant control lines of the same genetic backgrounds were performed using two-tailed Mann–Whitney *U*-tests, as described for Fig. 4. *P<0.05, **P<0.01 and ***P<0.001.

Drug assays with gene-edited control parasites carrying only silent ZFN binding-site mutations showed no change in IC_50_ values relative to the parental lines (see NF10^*mdr1*-YF-1^ versus NF10 and KC5^*mdr1*-YF-1^ versus KC5; Figs 4 and 5). Mean±s.e.m. IC_50_ and IC_90_ values, numbers of independent assays performed and Mann–Whitney tests for statistical significance are listed in Supplementary Tables 3–6. Control assays with the antimalarial drug atovaquone, which targets the mitochondrial bc1 complex^33^, showed no changes in IC_50_ values between any of the *pfmdr1* recombinant lines and their parental controls (Supplementary Fig. 1).

### PfMDR1 N86Y increases susceptibility to arylaminoalcohols and DHA

The most striking phenotype observed with the replacement of N86Y with the wild-type N86 residue was a significant increase in the IC_50_ and IC_90_ values for LMF, MFQ and DHA (Fig. 4 and Supplementary Tables 3–6). The change to N86 resulted in a three- to fourfold increase in the IC_50_ values for LMF and MFQ in both the NF10 and KC5 backgrounds (*P*<0.001, Mann–Whitney *U*-test).

In the case of DHA, the change to N86 resulted in ∼1.5-fold increased IC_50_ values in both backgrounds (*P*<0.05). NF10 recombinant and parental lines were also subjected to *in vitro* survival assays, whereby highly synchronized early ring stages (0–3 h post-invasion) or trophozoites (18–21 h post-invasion) were exposed to 700 nM DHA for 6 h, followed by washing to remove drug and assessment of parasite growth 66 h later^34^. Results from three independent RSA_0–3h_ and TSA_18–21h_ assays found no significant difference between the various PfMDR1 haplotypes (Supplementary Fig. 2). As a control, we included the Cam3.II^R539T^ line, which expresses the K13 R539T mutation that confers *in vitro* ring-stage resistance to DHA^7^. These results provide evidence that the differences observed in the 72 h *in vitro* inhibition assays did not translate into survival benefits on short-term exposure to a concentration of DHA.

### PfMDR1 N86Y decreases susceptibility to CQ and md-ADQ

Our assays revealed that the parental NF10 line was less sensitive to CQ, md-CQ and QN but was more sensitive to md-ADQ when compared with the parental KC5 line. This is consistent with earlier studies of the 7G8 × GB4 cross^19^ that attributed these changes to the combined effects of PfCRT and PfMDR1. In the case of the NF10 and KC5 progeny that harbour the same PfMDR1 YF haplotype, these differences in drug response can be attributed primarily to mutations in PfCRT (CVIET and SVMNT, respectively). The replacement of N86Y with the wild-type N86 residue resulted in increased susceptibility to CQ, md-CQ and md-ADQ with the greatest difference being observed in the NF10 recombinant parasites (Fig. 4).

For QN and PPQ, increased susceptibility was also observed on replacement of the N86Y to N86 in the NF10 line. However, this difference reached significance in only one of the NF and NY recombinant clones (NF-2 and NY-2) for QN (*P*<0.05, Mann–Whitney *U*-test; Fig. 5) and only in the NY recombinant line for PPQ (*P*<0.01; Fig. 4). We also investigated whether the PfMDR1 N86Y and Y184F mutations affected the accumulation of radiolabelled CQ, QN or ADQ in recombinant NF10 parasites. These assays revealed no statistically significant effect of the N-terminal PfMDR1 mutations on the accumulation of these drugs by trophozoite-infected RBCs (Supplementary Fig. 3).

### PfMDR1 Y184F has a minor impact on drug response

Compared with the N86Y mutation, the Y184F mutation in PfMDR1 appears to have a weaker association with antimalarial effectiveness *in vivo*^13^,^35^. Consistent with these observations, we report that differences in the antimalarial IC_50_ values between parasite lines expressing the PfMDR1 184 wild-type or mutant residue were dependent on the status of residue 86, the class of drug and the *pfcrt* genetic background (Figs 4 and 5, and Supplementary Tables 3 and 4). In lines expressing N86Y, the presence of Y or F at position 184 had no detectable influence on parasite susceptibility to the drugs tested (*P*>0.1, Mann–Whitney *U*-test). In parasites with N86, isogenic Y184F and Y184 lines were generally very similar in their IC_50_ values for most drugs, with some exceptions presented below.

In the KC5 background, clones expressing the PfMDR1 NF haplotype presented slightly reduced IC_50_ values for LMF and MFQ when compared with the NY clones (Fig. 4 and Supplementary Table 4). In the NF10 background, the PfMDR1 NF clones exhibited higher IC_50_ values relative to the NY clones for CQ and PPQ, with significance achieved in one of the recombinant clones tested (Supplementary Table 3). As discussed below, this possible decrease in PPQ susceptibility in parasites expressing the PfMDR1 NF haplotype in the CVIET PfCRT background is consistent with the high prevalence of NF parasites in Cambodia, where PPQ is widely used.

In absolute numbers, our data revealed changes in susceptibility that remained within commonly employed cutoffs of resistance for CQ^36^ (IC_50_ values above 80 nM) or sensitivity for MFQ^37^ (IC_50_ values below 15 nM). Nevertheless, drug responses in our genome-edited parasites presented a notable fold change (up to fourfold for some of the drugs tested). Although these relative changes might not suffice to cause clinical failure, they may contribute to clinical outcome, in conjunction with other infection variables.

### PfMDR1 F1226Y shows no impact on *in vitro* drug response

Recent studies have identified nearly 50% prevalence of the PfMDR1 F1226Y mutation in Thailand and Cambodia, and suggested its contribution to altered antimalarial drug susceptibilities^38^,^39^. To examine the role of this mutation *in vitro*, we employed a single cross-over-based allelic exchange strategy to introduce the PfMDR1 F1226Y mutation into the CQ-resistant K1 strain (obtained from Thailand in the early 1980s), which harbours a single copy of *pfmdr1* with YYSNFD at positions 86, 184, 1034, 1042, 1226 and 1246 (Supplementary Fig. 4). Phenotypic assessment of the parental strain and the F1226Y recombinant lines revealed no difference in the IC_50_ values for any of the drugs tested (LMF, MFQ, ART, AS, PPQ, CQ, md-ADQ and QN; Supplementary Table 7). Further studies will be required to assess whether this mutation might have an impact on drug susceptibilities in other Asian parasites, especially given that these no longer harbour the N86Y mutation and are often *pfmdr1* multicopy (Fig. 2).

## Discussion

Our study of genetically engineered *P. falciparum* lines with isogenic controls provides direct evidence that the PfMDR1 mutation N86Y modulates parasite susceptibility to a wide range of first-line antimalarials. We demonstrate that the N86Y mutation increases parasite susceptibility to LMF, MFQ and DHA, and conversely augments resistance to md-ADQ and CQ. The PfMDR1 Y184F mutation had a notably lesser impact in our isogenic lines, with evidence of decreasing *P. falciparum* susceptibility to PPQ when paired with the N86 residue. These data, which resolve long-standing questions about the role of PfMDR1, provide a foundation for improved molecular surveillance of antimalarial drug resistance and help inform region-specific decisions on which ACTs to employ.

The most striking effect of N86Y was on susceptibility to LMF and MFQ, for which parasites engineered to express wild-type N86 showed three- to fourfold higher drug IC_50_ values compared with the mutant N86Y residue. These data confirm genotyping and drug association studies performed with African and Asian isolates^40^,^41^, and are consistent with reports that the N86 allele predominates in recurrent infections following treatment with ATM+LMF^13^,^42^. Indeed, a recent meta-analysis of 31 clinical trials revealed that patients infected with N86 parasites had a fivefold greater risk of parasite recrudescence following ATM+LMF treatment compared with those infected with N86Y parasites^15^. Those data illustrate the utility of the *in vitro* data generated herein in identifying susceptibility shifts that correlate with changes in clinical outcomes.

The N86Y mutation was also observed to alter the parasite response to DHA, with ∼1.5-fold decreased IC_50_ values compared with isogenic N86 parasites. Previous *in vitro* studies on laboratory strains and clinical isolates have also noted cross-resistance between MFQ, LMF and ART derivatives^23^,^24^,^35^. This differs from the ART resistance mediated by mutations in K13, which manifests as reduced ring-stage susceptibility to DHA^6^,^7^. The IC_50_ shifts observed here are likely to reflect a role for *pfmdr1* in reducing parasite susceptibility at the trophozoite stage when haemoglobin degradation is the most active, producing high intracellular concentrations of the ART-activating ferrous iron that resides within haem^43^.

**Figure 6:**
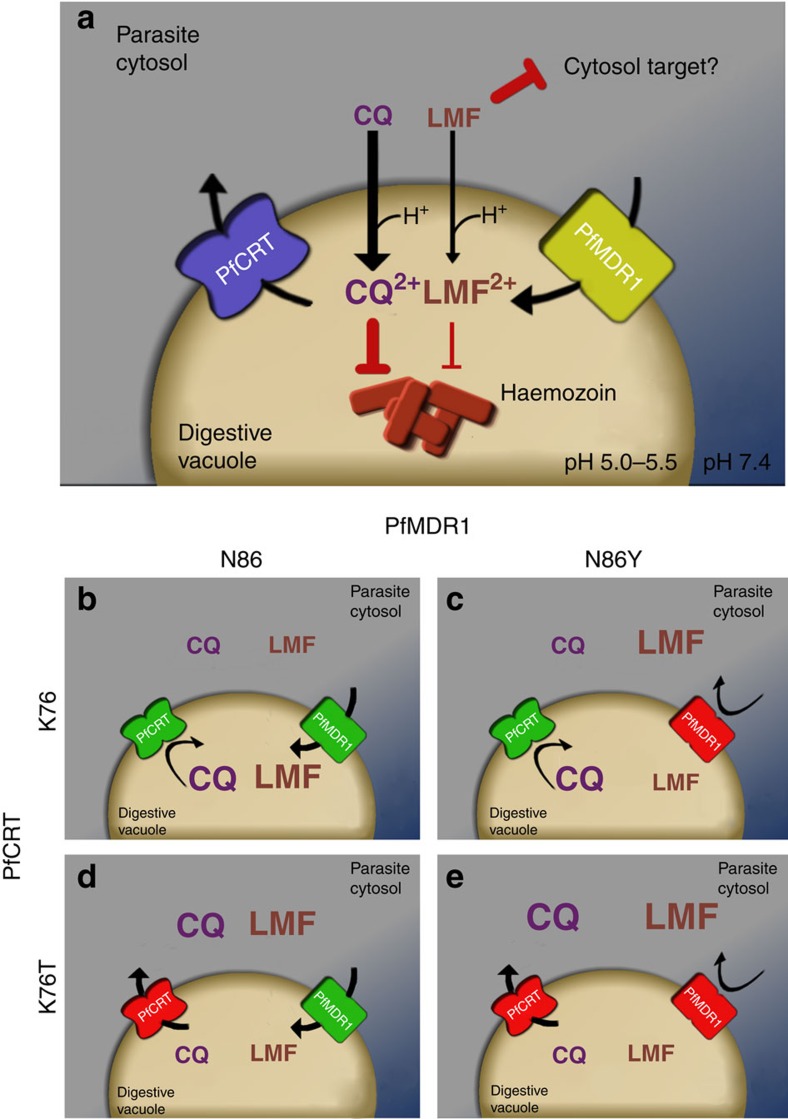
Proposed roles for PfMDR1 and PfCRT in the *P. falciparum* response to CQ and LMF. (**a**) CQ (a weak base) and the hydrophobic drug LMF are thought to enter the DV via simple diffusion across the membrane. Within the DV, CQ is thought to trigger parasite killing by binding to reactive haem moieties and preventing their biomineralization into chemically inert haemozoin crystals^67^. LMF also shows moderate binding to haem^2^; however, its main site of action is suspected to be outside of the DV. It is thought that PfCRT facilitates the efflux of drugs from the DV, and that PfMDR1 transports drugs into the DV^3^,^68^. (**b**–**e**) Point mutations in these transporters are known to differentially impact parasite susceptibility to CQ and LMF. The N86Y mutation is thought to ablate PfMDR1-mediated CQ transport activity^22^, but the resulting reduction in the rate of CQ influx is insufficient to confer CQ resistance in parasites that carry wild-type PfCRT (designated as K76)^47^ (**b**,**c**). CQ resistance-conferring isoforms of PfCRT (designated K76T) mediate the efflux of CQ from the DV^68^,^69^,^70^. The reduced rate of CQ influx in PfMDR1 N86Y parasites may further decrease CQ concentrations in the DV and thus augment CQ resistance. However, this effect is only evident in parasites expressing a high-capacity transporter of CQ (for example, GB4 PfCRT)^47^. This is perhaps because the loss of CQ influx via PfMDR1 causes a substantial decrease in the time taken to reach sub-lethal levels of CQ in the DV only when the drug is being extruded from the DV via K76T PfCRT at a relatively high rate. (**e**) LMF might also be a substrate of PfMDR1 N86 (**b**,**d**) and the introduction of N86Y (**c**,**e**) might likewise reduce the capacity of PfMDR1 to import LMF into the DV. In this scenario, the heightened susceptibility of CQ-resistant N86Y PfMDR1 parasites to LMF could be due to the increased accumulation of LMF in the cytosol (where its main antimalarial target may be located). The relative sizes of the CQ and LMF text in these panels reflect the predicted distribution of the drugs between the DV and cytosol.

In contrast to our finding that the N86Y mutation increases parasite susceptibility to LMF, MFQ and DHA, we observed that this mutation decreases parasite susceptibility to CQ, md-CQ and md-ADQ in the NF10 background. CQ resistance has earlier been proposed to have some degree of association with the N86Y mutation, both in South American and Asian/African backgrounds^44^. Previous studies with patient isolates have also linked the N86Y mutation to reduced parasite susceptibility to ADQ^45^,^46^. Our data reveal that the effect of the N86Y mutation on parasite response to CQ and md-CQ is significant in parasites expressing the CVIET PfCRT variant. This effect, however, is modest in parasites expressing the SVMNT PfCRT variant. The latter observation is probably due, at least in part, to differences in the kinetics of PfCRT-mediated CQ and md-CQ transport between the high-capacity CVIET transporters and the low-capacity SVMNT transporters^47^. This possibility is discussed in more detail below and in Fig. 6.

The involvement of residue 86 in QN resistance has been controversial^48^,^49^. Our results indicate that residue 86 of PfMDR1 plays a minor, background-specific role in QN susceptibility, with N86Y producing a slight increase in QN resistance only in NF10 parasites (that carry CVIET PfCRT). Mutant PfCRT is known to contribute to QN resistance, although the full repertoire of parasite determinants remains to be identified^48^,^50^,^51^.

Whereas the N86Y mutation affected the susceptibility of parasites to CQ and md-ADQ (and to a lesser extent QN), N86 and N86Y clones in the NF10 background showed no discernible difference in their accumulation of radiolabelled CQ, ADQ or QN (Supplementary Fig. 3). This suggests that if PfMDR1-mediated drug transport was occurring in parasites expressing a given PfMDR1 variant, the impact on drug accumulation was modest and beyond the limits of assay detection. Our data recall a prior analysis of the progeny of the GB4 × 7G8 genetic cross, which found that *pfmdr1* alleles could affect parasite drug susceptibility (as quantified using IC_50_ assays) without significantly altering levels of drug accumulation^51^. These findings suggest that polymorphisms within PfMDR1 and PfCRT can contribute to resistance phenotypes through mechanisms based not only on regulating drug accumulation inside the DV. One hypothesis, for which supporting evidence exists^52^,^53^,^54^, is that these antimalarials might bind these transporters directly and inhibit their physiological function. This mode of action would be in addition to these drugs interfering with reactive haem detoxification through interactions with haem^2^.

Our studies also revealed only a minimal impact of the Y184F mutation on antimalarial drug susceptibilities. In the NF10 background harbouring the predominant Asian/African *pfcrt* allele, there was a decreased susceptibility to PPQ in Y184F parasites, which was observed in the presence of the N86 residue. Structural models for PfMDR1 place residue 184 in the third transmembrane domain and it has been hypothesized that the Y184F mutation might affect the kinetics of PfMDR1-mediated drug transport^22^,^55^. Transport studies in *Xenopus* oocytes have also provided evidence of an effect of both the Y184F and N86Y mutations on the ability of PfMDR1 to transport different drugs^22^,^55^. Studies with proteoliposomes also provide evidence that PfMDR1 or PfCRT isoforms can differentially bind quinoline drugs^52^.

Previous studies have used traditional transfection-based approaches to explore the effects of the C-terminal mutations S1034C, N1042D and D1246Y on parasite drug responses^23^,^24^. These C-terminal PfMDR1 mutations, which are more frequent in low-transmission areas in South America than in Asia or Africa (as obtained from www.malariagen.net/pf3k), were found to influence parasite susceptibility to MFQ, QN and ART. These mutations also had an impact on the parasite's response to CQ in some genetic backgrounds^23^,^24^. Studies with isogenic transfectant lines have also revealed that an increase in *pfmdr1* copy number, which is very rare in Africa but which occurs relatively frequently in parts of Southeast Asia, reduces parasite susceptibility to MFQ, LMF, QN and ART^56^. Our study now elucidates the roles of the other two major PfMDR1 polymorphisms, N86Y and Y184F, which are the most prevalent variations in Africa. Of note, recent studies from Uganda have observed selection for PfMDR1 wild-type N86 and D1246 in patients treated with ATM+LMF, contrasting with selection of the mutations N86Y and D1246Y in patients receiving AS+ADQ^57^. Our evidence that N86Y increases parasite susceptibility to MFQ and LMF also helps explain why this mutation is very rarely observed in Thai parasites that have been under selection with these drugs, and that often carry multicopy *pfmdr1*, which has the opposite effect of reducing susceptibility (Fig. 4 and Supplementary Table 1). Our efficient gene-editing method now enables the examination of additional PfMDR1 polymorphisms that have recently been identified in field isolates, presumably as a result of the change in the past decade from CQ to ACTs.

Our study lends further support to the notion that the parasite genetic background is an important factor in determining the impact of PfMDR1 polymorphisms on parasite drug response. Studies with progeny of the GB4 × 7G8 cross have implicated PfCRT as a major driver of these divergent responses^19^. Evidence from heterologous expression studies in *Xenopus* oocytes suggest that GB4 PfCRT is a relatively high-capacity transporter of CQ, whereas the 7G8 isoform has a much lower capacity (but somewhat higher affinity) for CQ transport^47^. GB4 PfCRT might therefore be effective in decreasing high concentrations of CQ within the DV to sub-lethal levels. In this scenario the simultaneous action of an additional mechanism for reducing CQ accumulation—for example, mutations in PfMDR1 that ablate its ability to import CQ into the DV—could translate into further increases in the parasite's resistance to CQ that are attributable to the PfMDR1 variant. By contrast, 7G8 PfCRT might act more slowly to reduce CQ concentrations to sub-lethal levels. During that time the reduced CQ transport activity of the PfMDR1 variant might be mostly or completely masked, because, even with the reduced rate of CQ influx into the DV, the vacuolar concentration of CQ would be such that parasite growth and/or viability is still significantly reduced. This is but one possible mechanistic explanation for our findings (Fig. 6) and further studies are required to delineate the mechanisms by which different variants of PfCRT and PfMDR1 combine to affect drug susceptibility.

The recent availability of thousands of *P. falciparum* genomes now makes it possible to also examine regional associations between PfMDR1 and PfCRT haplotypes (Table 1 and Supplementary Table 1). In Africa, nearly 70% of the recently sequenced isolates carried the CQ-sensitive PfCRT CVMNK (residue 72–76) haplotype, with the remainder carrying the CQ-resistant CVIET haplotype. This recent resurgence of CQ-sensitive isolates in Africa that harbour wild-type *pfcrt* emphasizes the value of expanding *pfmdr1* gene-editing studies to those parasites. In Africa, the most abundant PfMDR1 N-terminal haplotypes were NF and NY. In Asia, the PfCRT haplotypes were almost all CVIET (80%) with the remainder being the CVIDT variant that was observed to have a minimal fitness cost compared with CVIET parasites^58^. Asian parasites were almost all NF or NY, with the latter associating the most with the CVIDT haplotype.

Our findings also provide new insights into the ways in which drug pressures have shaped different parasite populations. In Thailand, the reliance on AS+MFQ^59^ might explain why fewer than 1% of Thai isolates harbour the PfMDR1 N86Y mutation (Supplementary Table 1), which based on our data increases parasite susceptibility to both MFQ and DHA, and which therefore would be selected against. Indeed, MFQ has been used widely throughout Southeast Asia, which could explain the overall rarity of the N86Y mutation in this region. Bangladesh is an exception in that it has a significant proportion (22%) of N86Y isolates. This prevalence may reflect the fact that the national malaria control programme in Bangladesh administers CQ and QN in addition to ATM+LMF^60^. We find that PfMDR1 N86Y decreases parasite susceptibility to both CQ and QN, suggesting its active selection in this country. The N86Y mutation also decreases parasite susceptibility to md-ADQ, which could explain why this mutation is commonly observed in West Africa where AS+ADQ is frequently used. Malawi, on the other hand, relies on ATM+LMF as its first-line ACT, and in this east African country <2% of parasite isolates have the LMF-sensitizing N86Y mutation. Given the rapid changes in antimalarial drug use in Africa over the past 15 years, it will be of interest to assess changes over time in *pfmdr1* positions 86, 184 and 1246. The currently available genome data sets, however, do not currently permit this chronological analysis. Regarding PfMDR1 184, Laos is the only Southeast Asian country shown in Fig. 2 that employs ATM+LMF as its sole ACT, with the other countries having a treatment policy that includes the use of DHA+PPQ^61^. In light of our finding that the NF haplotype is associated with slightly decreased susceptibility to PPQ in parasites harbouring the Southeast Asian/African CVIET variant of PfCRT, we propose that the difference in treatment policies explains the rarity of the Y184F mutation in Laos (<4% of isolates) and its elevated frequency (>20% of isolates) in the other Southeast Asian countries that more commonly use DHA+PPQ. Based on our combined phenotypic and mapping data, we propose that an optimal policy for ACT usage would entail the use of LMF in areas with a high proportion of N86Y, ADQ in areas where N86 is prevalent and PPQ in areas with a high frequency of NY haplotype. The competing selective forces of LMF and ADQ, both on PfMDR1 and PfCRT^15^,^62^, also support scenarios of dual implementation of ATM+LMF and AS+ADQ, to suppress the emergence of high-grade multidrug resistance.

## Methods

### Analysis of the Pf3k *P. falciparum* genome data set

Genome analysis used data generated by the Pf3k project (www.malariagen.net/pf3k)^8^,^29^. Assembly files were downloaded from ftp://ngs.sanger.ac.uk/production/pf3k/release_3/, a publically accessible database. SNPs were manually extracted using the annotated Pf3D7 genome version 9.2 (the same used to assemble the field isolate genomes) and a custom Perl script used to call each base, its sample depth and allelic balance. Indels were not considered. The output was inserted as a table into a MySQL database that contained Pf3k metadata (including isolate origins and general mapping quality values). Using bash shell-MySQL connectors, we retrieved PfMDR1 (PF3D7_0523000) haplotype data from the database in an ordered list (separated by geographic region) on a per-genome basis. Haplotypes for amino acids 86 and 184 were counted and visualized in Excel. Copy number variations (CNVs) were assessed using the programme BICSeq^63^, incorporated in a shell script to permit parallel processing. Analysis was performed iteratively against several reference genomes to find the closest match for each field isolate. These included the West African laboratory strain 3D7, the Cambodian isolate PH0164-C and the Senegalese isolate SenT087.09. PH0164-C has a Southeast Asian PfCRT haplotype (eight mutations), no *pfmdr1* or *k13* mutations and no known *pfmdr1* CNVs. SenT087.09 has no CNVs, is wild-type for *pfcrt* and *pfmdr1*, and carries a triple mutant *pfdhfr* (encoding N51I, C59L and S108N) that is common in Africa and confers pyrimethamine resistance. Default parameters were set to a bin size of 300 nucleotides, a window size of 600 nucleotides and a smoothing factor of 12. To plot the distribution of CNVs, values were combined per country and separated by non-CNV-harbouring isolates and CNV-harbouring isolates of a certain haplotype. The geographical map was downloaded using free spatial data from DIVA-GIS (http://diva-gis.org) and imported into QGIS 2.10 (http://qgis.org/en/site) to be merged with the geo-referenced data. This map was overlaid with the previously calculated haplotypes as pie charts.

### Plasmid construction

CompoZr Custom ZFNs were purchased from Sigma-Aldrich (St. Louis, Missouri, USA) to induce a double-strand break in the 5′-end of the *pfmdr1* coding sequence. Two ZFNs (left and right), binding to adjacent sequences on opposite strands of the DNA helix, are required to induce the double-strand break. These were supplied on separate plasmids. The ZFN pair was designed to bind to the sequence 5′-GGTAACCTCAGTatcaaAGAAGAGGTTGAAAAAGA-3′ (the DNA cut site is shown in lower case letters and is situated 42 bp downstream of the *pfmdr1* start codon). The plasmid encoding the right ZFN also encoded the 2A ‘ribosome skip' peptide, which enables polycistronic expression of 2A-linked genes^26^. The two plasmids encoding the ZFNs were digested with BglII and XhoI, and combined to yield an intermediate plasmid with 2A-linked ZFN sequences. The ZFN fusion was then digested with NheI and XhoI, and subcloned downstream of a calmodulin (PF14_0323) promoter and upstream of the *hsp86* (PF07_0030) 3′-untranslated region in a pDC2-based vector with the h*dhfr* selectable marker^26^, yielding the plasmid pZFN^*pfmdr1*^-h*dhfr*.

A 2.4-kb donor sequence, encompassing 555 bp of the *pfmdr1* 5′-untranslated region and 1,805 bp of the *pfmdr1* coding sequence, was amplified from NF54 genomic DNA with the primers p1+p2 (Supplementary Table 8) and inserted into the BstAPI and AatII sites of pZFN^*pfmdr1*^-h*dhfr* to yield the editing plasmid p*mdr1*. To prevent edited genomes from being further cleaved, three silent mutations were engineered into the donor sequence at the ZFN-binding site via site-directed mutagenesis (QuikChange Multi SDM Kit; Agilent Technologies, Santa Clara, California, USA) using the primers p9+p10. The donor template, containing the three silent mutations, was further mutated as required to introduce point mutations at *pfmdr1* codons 86 and 184 (using the primer pairs p11+p12 or p13+p14), yielding four transfection plasmids (Fig. 3).

### Parasite culture and transfection

*P. falciparum* NF10 and KC5 clones (kindly provided by Dr Thomas E. Wellems, NIH) were maintained at ∼3% haematocrit with human O^+^ RBCs (Interstate Blood Bank, Memphis, TN, USA) in RPMI-1640 supplemented with 2 g l^−1^ sodium bicarbonate, 2 mM L-glutamine, 25 mM HEPES, 10 μg ml^−1^ gentamycin, 50 μM hypoxanthine and 0.5% (w/v) AlbuMAXII (Invitrogen, Waltham, Massachusetts, USA). They were maintained at 37 °C in an airtight environment flushed with 5% O_2_/5% CO_2_/90% N_2_. Ring-stage cultures at 5–8% parasitaemia were electroporated with ∼70 μg of purified plasmid DNA eluted in 1 × Cytomix, as described^26^.

Four different plasmids expressing the PfMDR1 86/184 residues (YF, YY, NF or NY) were used to electroporate NF10 and KC5 cultures. The plasmids were designed to modify *pfmdr1* codons 86 and 184, and to integrate three silent mutations at the ZFN-binding site. To enable transient ZFN expression and consequent homology repair, WR99210 (2.5 nM; Jacobus Pharmaceuticals, Plainsboro, New Jersey, USA) was applied to transfected cultures 24 h postelectroporation and maintained for 4–6 days. We cloned edited parasites by limiting dilution in 96-well plates.

### DNA analysis

Clones from transfected NF10 and KC5 parasites were checked for ZFN-mediated editing by PCR amplification from genomic DNA using the KAPA Blood PCR Kit B (KAPA Biosystems, Wilmington, Massachusetts, USA) and the primers p3 (located upstream of the *pfmdr1* start codon and not in the plasmid ‘donor sequence') and p4 (Supplementary Table 8). The silent mutations introduced into the donor sequence create an SmlI restriction site, which was used to screen PCR products. Amplified fragments containing the silent mutations were sequenced to confirm editing at codons 86 and 184. Successfully edited clones were expanded for further analysis. *pfmdr1* copy number analysis was performed using a published Taqman-based quantification assay^35^. 3D7 and FCB were used as control parasites, as they harbour 1 and 2 copies of *pfmdr1*, respectively (Supplementary Table 2).

### *In vitro* antimalarial drug assays

Drug inhibitory assays were performed using a flow cytometry-based method described previously^64^, with some modifications. Assays were performed in flat-bottom 96-well plates, with each well containing 200 μl of cell suspension in culture medium, with or without the test compound as appropriate. The starting haematocrit was 1% and the starting parasitaemia (consisting of predominantly ring-stage parasites) was 0.1–0.2%. After 72 h incubation at 37 °C, samples were analysed by flow cytometry. Cells were stained with 1.6 μM Mito Tracker Deep Red and 2 × SYBR Green (Invitrogen) in 1 × PBS^64^. *In vitro* IC_50_ and IC_90_ values were calculated using nonlinear regression analysis. *In vitro* Ring-stage Survival Assay (RSA_0–3h_) and Trophozoite-stage Survival Assay (TSA_18–21h_) were carried out with 700 nM DHA as previously described^7^,^34^. In summary, NF10 recombinant and parental lines were sorbitol and Percoll synchronized and tested as 0–3 h post-invasion ring stages and 18–21 h post-invasion trophozoites. Parasites were adjusted to 1% parasitaemia and exposed to 700 nM DHA for 6 h. After this time, drug was removed by washing and parasites were cultured a further 66 h. Parasite viability was assessed by flow cytometry as stated above. RBCs (20,000 to 35,000) were counted in two separate wells per experiment, which was repeated on three separate occasions^7^. Percent survival was calculated as the parasitaemia in the drug-treated sample divided by the parasitaemia in the untreated sample × 100 (Supplementary Fig. 2).

### Drug accumulation assays

Assays were performed on mature, intact trophozoite-infected erythrocytes suspended in bicarbonate-free RPMI 1640 medium (supplemented with 25 mM HEPES, 10 mM glucose and 0.2 mM hypoxanthine, and adjusted to pH 7.4) at a parasitaemia of 3.7–9.1% and a haematocrit of 2%. The final concentrations of [^3^H] CQ (20 Ci mmol^−1^), [^3^H] QN (80 Ci mmol^−1^) and [^3^H] ADQ (15 Ci mmol^−1^) were 20, 20 and 40 nM, respectively. Labelled drugs were purchased from American Radiolabeled Chemicals. Assays were performed according to a protocol described previously^65^ with one modification: the incorporation of radiolabelled drug into the acid-insoluble pellets was also measured^66^ and values included in determinations of the amount of drug taken up by the cells. Accumulation studies were also attempted with radiolabelled LMF; however, a high level of variability was observed. This may have resulted from the poor solubility of LMF in one of the solutions used in the experiment and/or from the diffusion of LMF into the oil layer that was used to terminate drug accumulation (the cells are separated from the radiolabelled drug solution by centrifugation through oil).

### Statistical tests

All statistical analyses used non-parametric two-tailed Mann–Whitney *U*-tests (normal distribution not assumed; performed with GraphPad Prism Software).

## Additional information

**How to cite this article**: Veiga, M. I. *et al.* Globally prevalent PfMDR1 mutations modulate *Plasmodium falciparum* susceptibility to artemisinin-based combination therapies. *Nat. Commun.* 7:11553 doi: 10.1038/ncomms11553 (2016).

## Supplementary Material

Supplementary Information Supplementary figures 1-4, Supplementary tables 1-8

## Figures and Tables

**Figure 1:**
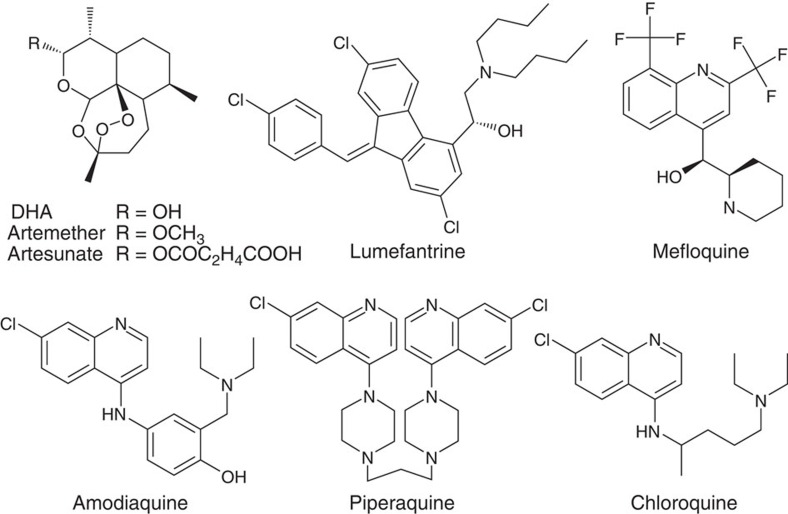
Chemical structures of antimalarials used in ACTs. Clinically used artemisinin derivatives are shown on the top left. DHA, dihydroartemisinin.

**Table 1:**
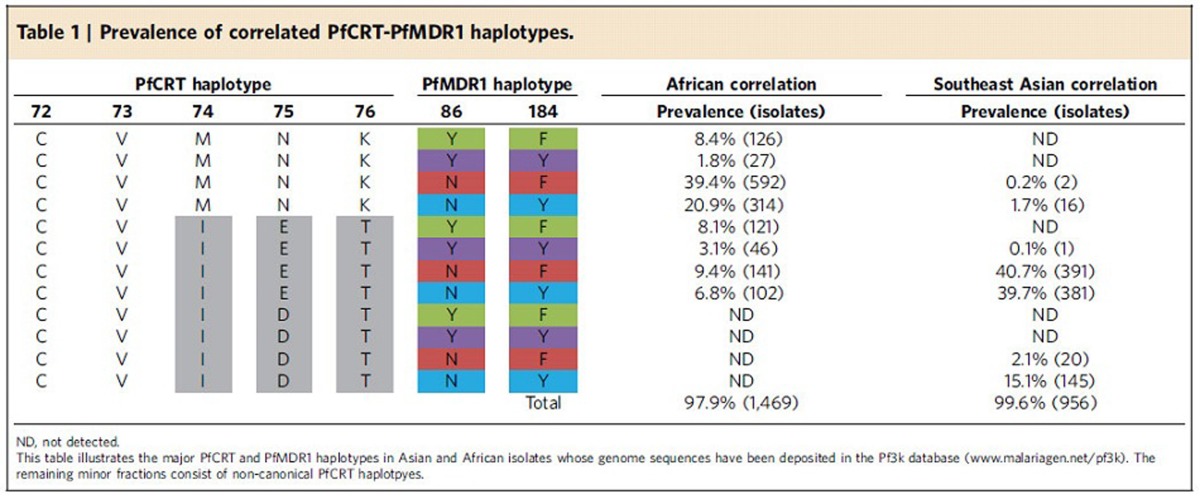
Prevalence of correlated PfCRT-PfMDR1 haplotypes.

**Table 2:**
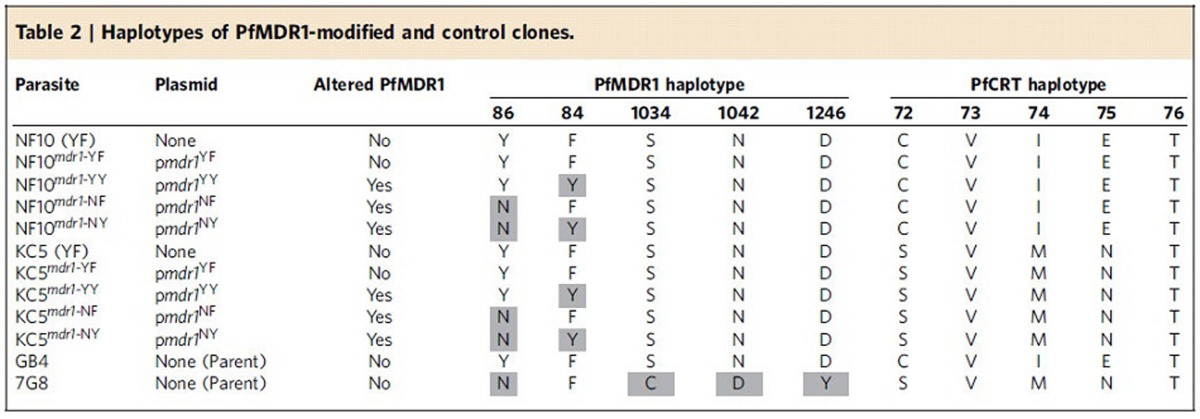
Haplotypes of PfMDR1-modified and control clones.
