# Supplementary material for: Globally prevalent PfMDR1 mutations modulate Plasmodium falciparum susceptibility to artemisinin-based combination therapies
Source: Nat Commun. 2016 May 18;7:11553. doi: 10.1038/ncomms11553 (PMC4873939; doi:10.1038/ncomms11553)
Supplement: Supplementary Information — Supplementary figures 1-4, Supplementary tables 1-8 [file ncomms11553-s1.pdf]

Supplementary Information

Globally prevalent PfMDR1 mutations modulate *Plasmodium falciparum* susceptibility to artemisinin-based combination therapies

M. Isabel Veiga, Satish K. Dhingra, Philipp P. Henrich, Judith Straimer, Nina Gnadig, Anne-Catrin Uhlemann, Rowena E. Martin, Adele M. Lehane, David A. Fidock

Supplementary Figure 1 | Atovaquone *in vitro* IC_{50} data with *pfmdr1*-modified and parental lines.

Supplementary Figure 2 | Ring-stage and Trophozoite-stage survival assays of *pfmdr1*-modified and parental NF10 lines**.**

Supplementary Figure 3 | *In vitro* drug accumulation profiles of *pfmdr1*-modified clones in the NF10 background and the recombinant control.

Supplementary Figure 4 | Allelic exchange strategy employed to introduce F1226Y into PfMDR1.

Supplementary Table 1 | Prevalence of PfMDR1 haplotypes at positions 86/184 and copy number variations and PfCRT residue 76 status in sampled malaria-endemic countries.

Supplementary Table 2 | Copy number analysis of *pfmdr1*-modified recombinant lines.

Supplementary Table 3 | Mean±SEM IC_{50} values (nM) of NF10 parental strain and *pfmdr1*-modified recombinant lines.

Supplementary Table 4 | Mean±SEM IC_{50} values (nM) of KC5 parental strain and *pfmdr1*-modified recombinant lines.

Supplementary Table 5 | Mean±SEM IC_{90} values (nM) of NF10 parental strain and *pfmdr1*-modified recombinant lines.

Supplementary Table 6 | Mean±SEM IC_{90} values (nM) of KC5 parental strain and *pfmdr1*-modified recombinant lines.

Supplementary Table 7 | Mean±SEM IC_{50} values of K1 parental strain and PfMDR1 F1226Y recombinant lines.

Supplementary Table 8 | List of oligonucleotides used in this study.

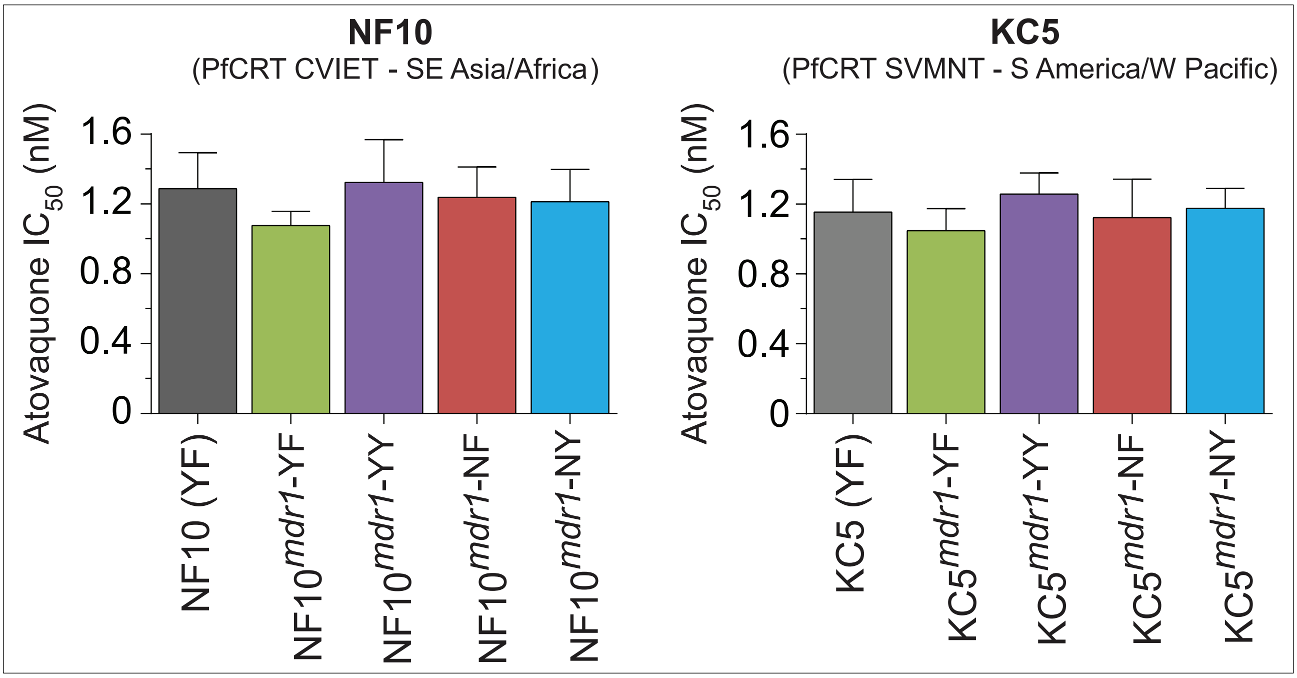

Supplementary Figure 1 | Atovaquone *in vitro* IC_{50} data with *pfmdr1*-modified and parental lines. Experiments were performed in duplicate on 5–7 independent occasions and IC_{50} values as presented as mean±SEM. No significant differences were observed between any *pfmdr1*-modified lines and their parental controls.

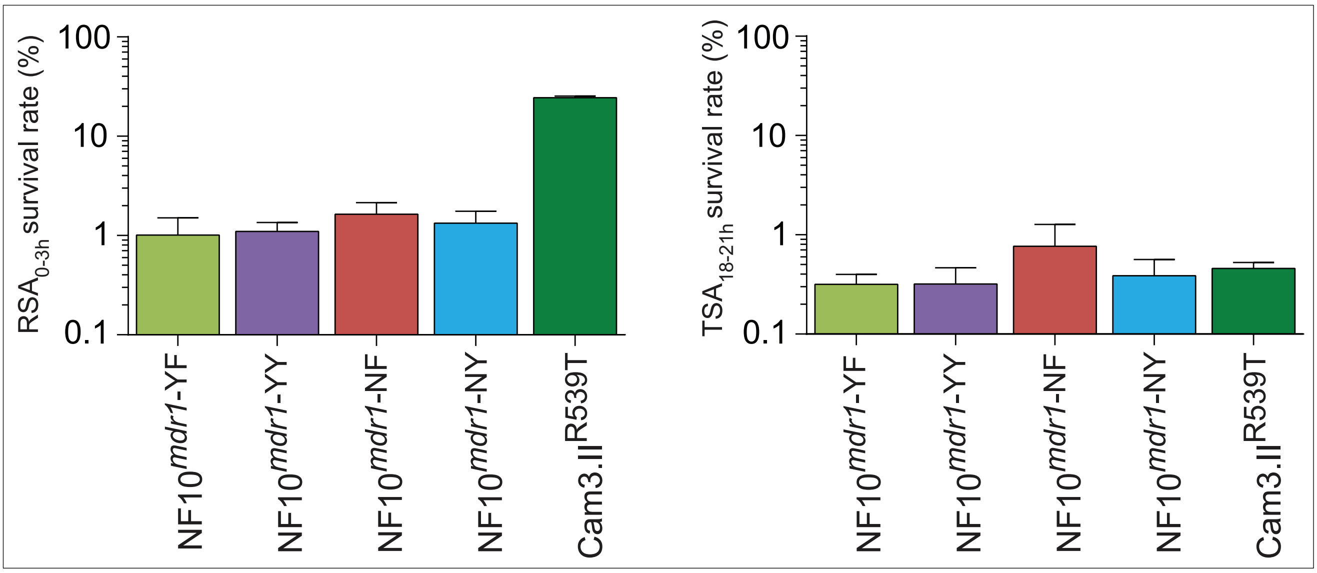

Supplementary Figure 2 | Ring-stage and Trophozoite-stage survival assays of *pfmdr1*-modified and parental NF10 lines. Data were generated using ring-stage and trophozoite-stage survival assays, prepared with synchronized parasites beginning 0-3 hr and 18-21 hr post-invasion (the RSA$_{0-3h}$ and TSA$_{18-21h}$) respectively. Parasites were exposed to 700 nM dihydroartemisinin for 6 hr, the drug removed by washing, and survival measured after 66 hr of further culture. The parasitemia of surviving parasites was determined by flow cytometry of 20,000–35,000 parasites stained with SYBR Green I and Mitotracker Deep Red. Assays were performed in duplicate on three separate occasions. Survival values (calculated for each line and assay as a percent survival of DMSO-control treated parasites from that same line) are shown as means ± SEM. Mann-Whitney *U* tests showed no significant difference between *pfmdr1*-modified lines and the parental NF10 control. As a positive control for the RSA$_{0-3h}$, we included the Cam3.II^{R539T} line, which expresses the K13 R539T mutation that confers *in vitro* ring-stage resistance to dihydroartemisinin.

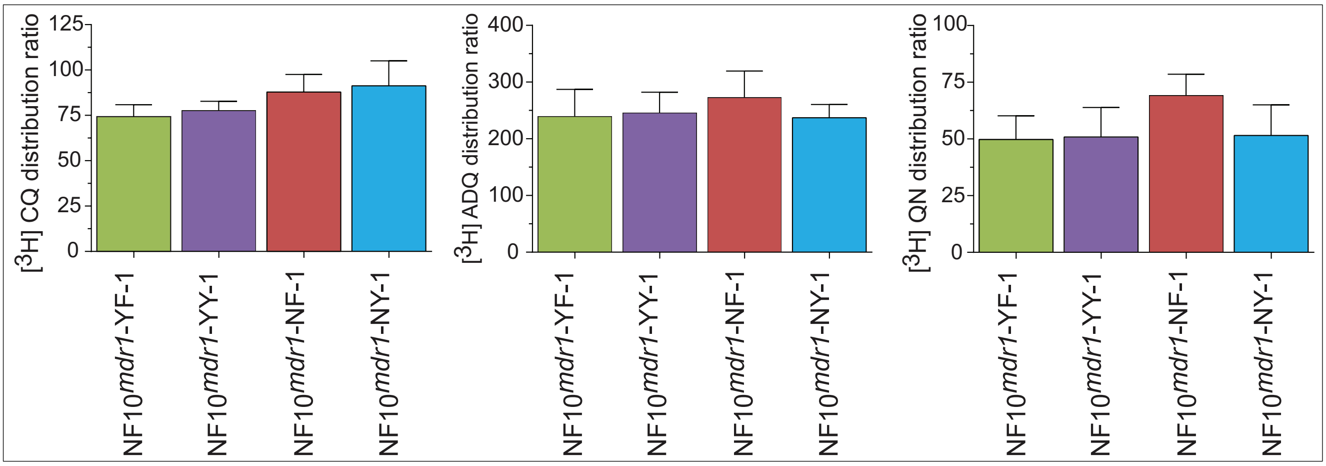

Supplementary Figure 3 | *In vitro* drug accumulation profiles of *pfmdr1*-modified clones in the NF10 background and the recombinant control. Accumulation of [^{3}H] CQ (tested at a concentration of 20 nM), [^{3}H] ADQ (40 nM), or [^{3}H] QN (20 nM) by erythrocytes infected with mature trophozoite-stage parasites. Data are shown for *pfmdr1*-modified lines generated in the NF10 background (clones analyzed: NF10$^{\textit{mdr1}\text{-YF-1}}$, NF10$^{\textit{mdr1}\text{-YY-1}}$, NF10$^{\textit{mdr1}\text{-NF-1}}$ and NF10$^{\textit{mdr1}\text{-NY-1}}$). [^{3}H]-labeled drug accumulation was measured over 1 hr at 37°C and was expressed as the 'distribution ratio', where the distribution ratio corresponds to the concentration of radiolabeled drug within the infected cells relative to the concentration in the extracellular medium. The data shown are mean + SEM of 4 independent experiments for QN and of 5 independent experiments for CQ and ADQ.

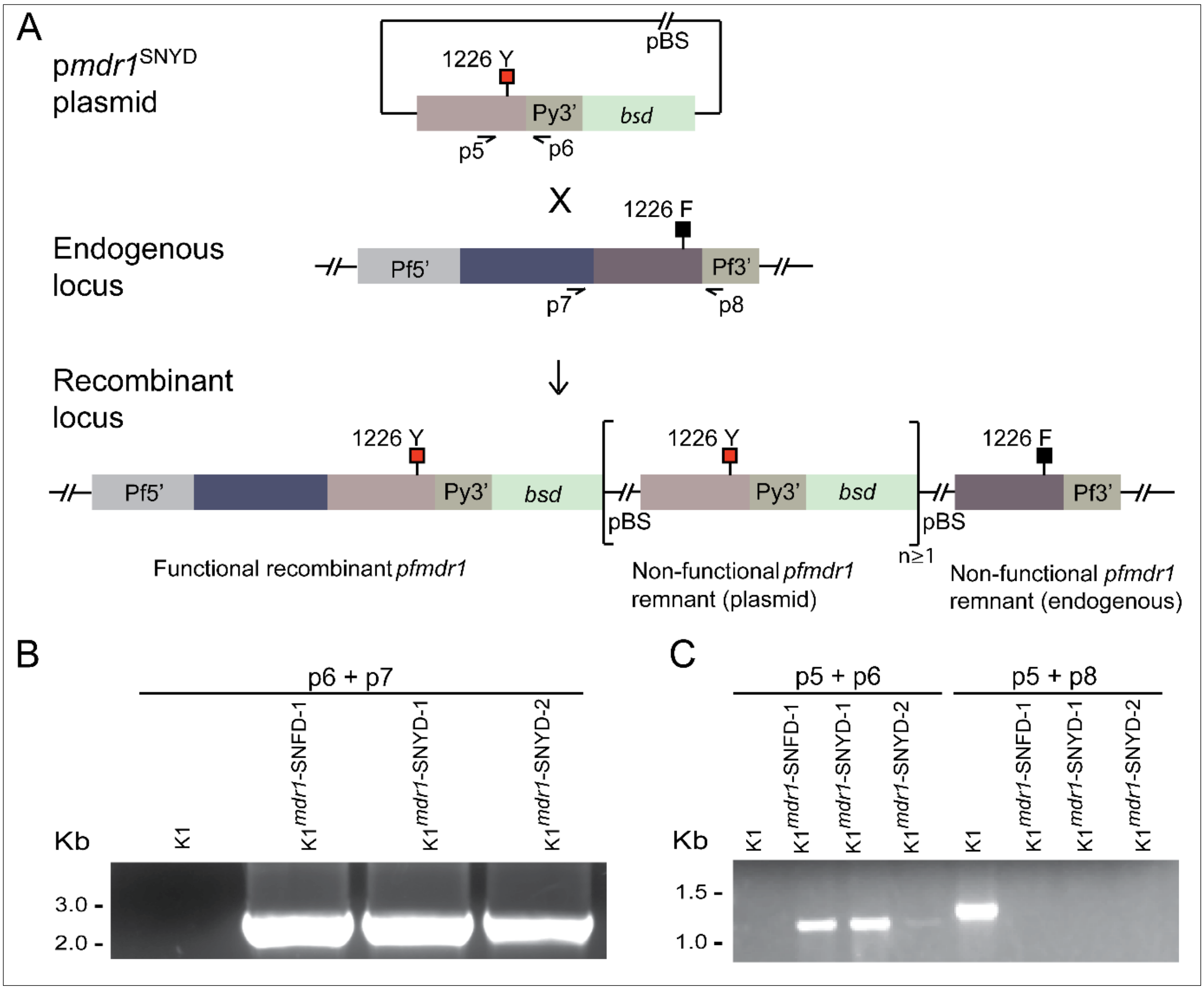

Supplementary Figure 4 | Allelic exchange strategy employed to introduce F1226Y into PfMDR1. (A) p*mdr1*^{SNYD} contains the last 2.1 kb of *pfmdr1* coding sequence, followed by a 0.7 kb fragment of the *P. yoelii mdr1* 3' UTR, and a blasticidin selectable marker (*bsd*). The *pfmdr1* sequence within the plasmid was modified by site-directed mutagenesis of the p*mdr1*^{SNFD} plasmid[24] using primers p15 and p16 to introduce the mutation encoding 1226Y. The endogenous K1 *pfmdr1* locus is flanked by *P. falciparum* 3' and 5' UTR elements and carries the wild-type F1226 allele. K1 parasites were transfected with p*mdr1*^{SNYD} to introduce the F1226Y mutation or with p*mdr1*^{SNFD} to obtain a recombinant control with an unchanged *pfmdr1* allele. Transfected parasites were selected with 1.25–2.0 µg/ml blasticidin. Integration of p*mdr1*^{SNYD} (or p*mdr1*^{SNFD}) into the endogenous *pfmdr1* locus, via single-site cross-over and homologous recombination, resulted in a recombinant locus that contained the functional recombinant *pfmdr1* locus and downstream non-functional *pfmdr1* remnants. The recombinant parasites were cloned by limiting dilution. **(B)** PCR-based screening of the edited clones K1$^{mdr1\text{-SNYD-1}}$ and K1$^{mdr1\text{-SNYD-2}}$, their recombinant control K1$^{mdr1\text{-SNFD}}$ and the parent K1. PCR was performed with primers p6 + p7, which were specific for the recombinant locus only and which together yielded no band in K1. The *pfmdr1* gene was fully sequenced for each line using PCR products obtained with primers p17 + p18 (5' end of

gene) and p6 + p7 (3’ end of gene; recombinant lines) or p7 + p8 (3’ end of gene; K1). The non-synonymous mutations identified (compared to the wild-type 3D7 sequence) were as predicted (N86Y for all lines and F1226Y for $K1^{mdr1\text{-SNYD-1}}$ and $K1^{mdr1\text{-SNYD-2}}$ only). **(C)** RT-PCR of $K1^{mdr1\text{-SNYD-1}}$ and $K1^{mdr1\text{-SNYD-2}}$, their recombinant control $K1^{mdr1\text{-SNFD}}$ and the parent K1, using primers p5 + p6 and p5 + p8. Primer pair p5 + p6 yielded a 1.0 kb c-DNA product with the *pfmdr1* recombinant lines only, whereas p5 + p8 yielded a 1.1 kb band with only the parental K1 strain and not with the recombinant clones.

Supplementary Table 1 | Prevalence of PfMDR1 haplotypes at positions 86/184 and copy number variations and PfCRT residue 76 status in sampled malaria-endemic countries.

Country	Region	Total Isolates	PfMDR1 haplotypes at positions 86/184 total number (percentage of total)				*pfmdr1* Copy Number Variation > 1.5-fold total number (percentage of total)						PfCRT status	
			YF	YY	NF	NY	No CNV	CNV >1.5x	YF + CNV	YY + CNV	NF + CNV	NY + CNV	K76	K76T
DR Congo	Africa	113	17 (15.0%)	35 (31.0%)	29 (25.7%)	32 (28.3%)	107 (94.7%)	6 (5.3%)	0	1 (0.9%)	3 (2.6%)	2 (1.8%)	33 (29.2%)	80 (70.8%)
Ghana	Africa	617	100 (16.2%)	23 (3.7%)	326 (52.8%)	168 (27.2%)	613 (99.4%)	4 (0.6%)	0	0	3 (0.5%)	1 (0.2%)	492 (79.7%)	125 (20.3%)
Guinea	Africa	100	29 (29.0%)	9 (9.0%)	38 (38.0%)	24 (24.0%)	100 (100%)	0	0	0	0	0	27 (27.0%)	73 (73.0%)
Malawi	Africa	369	4 (1.1%)	3 (0.8%)	193 (52.3%)	169 (45.8%)	369 (100%)	0	0	0	0	0	369 (100%)	0
Mali	Africa	96	31 (32.3%)	2 (2.1%)	38 (39.6%)	25 (26.0%)	96 (100%)	0	0	0	0	0	41 (42.7%)	55 (57.3%)
Nigeria	Africa	5	2 (40.0%)	0	2 (40.0%)	1 (20.0%)	5 (100%)	0	0	0	0	0	1 (20.0%)	4 (80.0%)
Senegal	Africa	137	17 (12.4%)	2 (1.5%)	71 (51.8%)	47 (34.3%)	135 (98.5%)	2 (1.5%)	0	0	0	2 (1.5%)	77 (56.2%)	60 (43.8%)
The Gambia	Africa	65	17 (26.2%)	1 (1.5%)	24 (36.9%)	23 (35.4%)	65 (100%)	0	0	0	0	0	19 (29.2%)	46 (70.8%)
Bangladesh	S. Asia	50	0	11 (22.0%)	10 (20.0%)	29 (58.0%)	47 (94.0%)	3 (6.0%)	0	0	0	3 (6.0%)	4 (8.0%)	46 (92.0%)
Cambodia	S.E. Asia	570	0	0	312 (54.7%)	258 (45.3%)	489 (85.8%)	81 (14.2%)	0	0	66 (11.6%)	15 (2.6%)	12 (2.1%)	558 (97.9%)
Laos	S.E. Asia	85	0	0	3 (3.5%)	82 (96.5%)	85 (100%)	0	0	0	0	0	4 (4.7%)	81 (95.3%)
Myanmar	S.E. Asia	60	0	0	24 (40.0%)	36 (60.0%)	53 (88.3%)	7 (11.7%)	0	0	3 (5.0%)	4 (6.7%)	0	60 (100%)
Thailand	S.E. Asia	148	0	1 (0.7%)	43 (29.0%)	104 (70.3%)	90 (60.8%)	58 (39.2%)	0	0	9 (6.1%)	49 (33.1%)	0	148 (100%)
Vietnam	S.E. Asia	97	0	0	22 (22.7%)	75 (77.3%)	91 (93.8%)	6 (6.2%)	0	0	4 (4.1%)	2 (2.1%)	2 (2.1%)	95 (97.9%)

This analysis used data generated by the Pf3k project (www.malariagen.net/pf3k). S. E., Southeast.

Supplementary Table 2 | Copy number analysis of *pfmdr1*-modified recombinant lines.

Parasite line	Mean estimate	Standard error	Calculated *pfmdr1* copy number	Number of independent repeats
NF10 (YF)	0.96	0.12	1	3
$NF10^{mdr1\text{-}YF}$	1.04	0.15	1	3
$NF10^{mdr1\text{-}YY}$	1.01	0.04	1	2
$NF10^{mdr1\text{-}NF}$	1.15	0.13	1	3
$NF10^{mdr1\text{-}NY}$	0.95	0.14	1	3
FCB	1.85	0.08	2	2
KC5 (YF)	0.97	0.27	1	3
$KC5^{mdr1\text{-}YF}$	0.92	0.10	1	4
$KC5^{mdr1\text{-}YY}$	1.09	0.13	1	4
$KC5^{mdr1\text{-}NF}$	0.94	0.11	1	4
$KC5^{mdr1\text{-}NY}$	0.89	0.16	1	4
FCB	1.88	0.05	2	4

pfmdr1 copy number was determined using a published Taqman assay (see Methods). n.d., not determined.

Supplementary Table 3 (page 1 of 2) | Mean±SEM IC_{50} values (nM) of NF10 parental strain and *pfmdr1*-modified recombinant lines.

Line	NF10 (YF)	NF10$^{mdr1\text{-YF-1}}$	NF10$^{mdr1\text{-YY-1}}$	NF10$^{mdr1\text{-YY-2}}$	NF10$^{mdr1\text{-NF-1}}$	NF10$^{mdr1\text{-NF-2}}$	NF10$^{mdr1\text{-NY-1}}$	NF10$^{mdr1\text{-NY-2}}$
**LMF IC_{50} (nM)**	**0.9 ± 0.1**	**0.9 ± 0.1**	**0.9 ± 0.1**	**0.9 ± 0.1**	**3.1 ± 0.4**	**2.7 ± 0.3**	**3.2 ± 0.4**	**3.1 ± 0.3**
# assays	7	7	7	7	5	5	6	6
p value								
NF10$^{mdr1\text{-YF-1}}$			0.81	0.81	0.0025	0.0025	0.0012	0.0012
NF10$^{mdr1\text{-YY-1}}$				0.90	0.0025	0.0025	0.0012	0.0012
NF10$^{mdr1\text{-YY-2}}$					0.0025	0.0025	0.0012	0.0012
NF10$^{mdr1\text{-NF-1}}$						0.55	0.66	0.93
NF10$^{mdr1\text{-NF-2}}$							0.33	0.43
NF10$^{mdr1\text{-NY-1}}$								0.94
**MFQ IC_{50} (nM)**	**3.9 ± 0.2**	**4.0 ± 0.3**	**4.1 ± 0.3**	**3.9 ± 0.2**	**12.8 ± 0.9**	**12.3 ± 1.0**	**12.5 ± 1.1**	**12.6 ± 1.1**
# assays	7	7	7	7	7	7	7	7
p value								
NF10$^{mdr1\text{-YF-1}}$			0.81	0.62	0.0006	0.0006	0.0006	0.0006
NF10$^{mdr1\text{-YY-1}}$				p>0.9999	0.0006	0.0006	0.0006	0.0006
NF10$^{mdr1\text{-YY-2}}$					0.0006	0.0006	0.0006	0.0006
NF10$^{mdr1\text{-NF-1}}$						0.62	0.62	0.90
NF10$^{mdr1\text{-NF-2}}$							0.81	p>0.9999
NF10$^{mdr1\text{-NY-1}}$								0.71
**DHA IC_{50} (nM)**	**0.60 ± 0.04**	**0.61 ± 0.05**	**0.62 ± 0.05**	**0.61 ± 0.03**	**0.93 ± 0.13**	**0.84 ± 0.12**	**0.97 ± 0.12**	**0.89 ± 0.09**
# assays	7	7	7	7	7	7	7	7
p value								
NF10$^{mdr1\text{-YF-1}}$			0.81	0.62	0.0070	0.04	0.0070	0.02
NF10$^{mdr1\text{-YY-1}}$				0.71	0.03	0.07	0.02	0.04
NF10$^{mdr1\text{-YY-2}}$					0.0070	0.04	0.0070	0.03
NF10$^{mdr1\text{-NF-1}}$						0.32	0.81	p>0.9999
NF10$^{mdr1\text{-NF-2}}$							0.26	0.32
NF10$^{mdr1\text{-NY-1}}$								0.81
**PPQ IC_{50} (nM)**	**41.8 ± 3.4**	**40.2 ± 2.5**	**37.4 ± 4.4**	**35.4 ± 3.3**	**35.4 ± 2.1**	**35.1 ± 1.0**	**31.4 ± 1.5**	**28.9 ± 1.0**
# assays	6	6	6	6	6	6	6	6
p value								
NF10$^{mdr1\text{-YF-1}}$			0.7	0.39	0.31	0.09	0.0087	0.0022
NF10$^{mdr1\text{-YY-1}}$				0.82	0.94	0.82	0.39	0.18
NF10$^{mdr1\text{-YY-2}}$					0.94	0.82	0.70	0.18
NF10$^{mdr1\text{-NF-1}}$						0.94	0.24	0.04
NF10$^{mdr1\text{-NF-2}}$							0.09	0.0087
NF10$^{mdr1\text{-NY-1}}$								0.39

Supplementary Table 3 (page 2 of 2) | Mean±SEM IC_{50} values (nM) of NF10 parental strain and *pfmdr1*-modified recombinant lines.

**CQ IC_{50} (nM)**	**347.1 ± 24.4**	**328.2 ± 29.7**	**331.1 ± 24.5**	**331.2 ± 21.1**	**216.2 ± 15.3**	**204.8 ± 12.1**	**186.6 ± 10.4**	**184.5 ± 6.6**
# assays	6	6	6	6	6	6	6	6
p value								
NF10$^{mdr1\text{-}YF\text{-}1}$			0.94	p>0.9999	0.04	0.04	0.04	0.03
NF10$^{mdr1\text{-}YY\text{-}1}$				p>0.9999	0.0043	0.0043	0.0022	0.0022
NF10$^{mdr1\text{-}YY\text{-}2}$					0.0043	0.0043	0.0022	0.0022
NF10$^{mdr1\text{-}NF\text{-}1}$						0.31	0.04	0.04
NF10$^{mdr1\text{-}NF\text{-}2}$							0.24	0.07
NF10$^{mdr1\text{-}NY\text{-}1}$								0.24
**md-CQ IC_{50} (nM)**	**1187.0 ± 97.6**	**1193.3 ± 84.5**	**1109.9 ± 106.2**	**1065.3 ± 79.8**	**881.2 ± 61.7**	**796.6 ± 33.5**	**780.4 ± 38.6**	**743.4 ± 35.4**
# assays	6	6	6	6	6	6	6	6
p value								
NF10$^{mdr1\text{-}YF\text{-}1}$			0.59	0.31	0.04	0.03	0.02	0.0087
NF10$^{mdr1\text{-}YY\text{-}1}$				0.82	0.31	0.07	0.07	0.0087
NF10$^{mdr1\text{-}YY\text{-}2}$					0.13	0.0087	0.0043	0.0022
NF10$^{mdr1\text{-}NF\text{-}1}$						0.18	0.09	0.04
NF10$^{mdr1\text{-}NF\text{-}2}$							0.94	0.49
NF10$^{mdr1\text{-}NY\text{-}1}$								0.31
**md-ADQ IC_{50} (nM)**	**76.8 ± 5.2**	**76.1 ± 5.0**	**76.8 ± 4.7**	**77.2 ± 3.8**	**47.1 ± 1.6**	**44.8 ± 0.6**	**45.0 ± 1.2**	**42.7 ± 0.6**
# assays	6	6	6	6	6	6	6	6
p value								
NF10$^{mdr1\text{-}YF\text{-}1}$			0.82	0.94	0.0022	0.0022	0.0022	0.0022
NF10$^{mdr1\text{-}YY\text{-}1}$				p>0.9999	0.0022	0.0022	0.0022	0.0022
NF10$^{mdr1\text{-}YY\text{-}2}$					0.0022	0.0022	0.0022	0.0022
NF10$^{mdr1\text{-}NF\text{-}1}$						0.31	0.70	0.04
NF10$^{mdr1\text{-}NF\text{-}2}$							0.59	0.09
NF10$^{mdr1\text{-}NY\text{-}1}$								0.18
**QN IC_{50} (nM)**	**121.5 ± 8.8**	**127.1 ± 6.1**	**137.6 ± 6.3**	**126.1 ± 3.2**	**103.6 ± 6.5**	**99.5 ± 6.3**	**107.2 ± 5.7**	**98.8 ± 6.7**
# assays	5	5	5	5	5	5	5	5
p value								
NF10$^{mdr1\text{-}YF\text{-}1}$			0.42	0.84	0.06	0.02	0.10	0.02
NF10$^{mdr1\text{-}YY\text{-}1}$				0.15	0.02	0.02	0.02	0.02
NF10$^{mdr1\text{-}YY\text{-}2}$					0.03	0.03	0.06	0.03
NF10$^{mdr1\text{-}NF\text{-}1}$						0.84	0.69	0.55
NF10$^{mdr1\text{-}NF\text{-}2}$							0.22	p>0.9999
NF10$^{mdr1\text{-}NY\text{-}1}$								0.55

IC_{50} values are expressed as mean ± SEM. (LMF) lumefantrine, (MFQ) mefloquine, (DHA) dihydroartemisinin, (PPQ) piperaquine, (CQ) chloroquine, (md-CQ) monodesethyl-chloroquine, (md-ADQ) monodesethyl-amodiaquine and (QN) quinine.

Statistical comparisons were made using Mann–Whitney U test.

Color code:				
	not significant	$p<0.05$	$p<0.01$	$p<0.001$

Supplementary Table 4 (page 1 of 2) | Mean±SEM IC_{50} values (nM) of KC5 parental strain and *pfmdr1*-modified recombinant lines.

Line		KC5 (YF)	$KC5^{mdr1\text{-}YF\text{-}1}$	$KC5^{mdr1\text{-}YY\text{-}1}$	$KC5^{mdr1\text{-}NF\text{-}1}$	$KC5^{mdr1\text{-}NF\text{-}2}$	$KC5^{mdr1\text{-}NY\text{-}1}$	$KC5^{mdr1\text{-}NY\text{-}2}$
**LMF IC_{50} (nM)**		**0.8 ± 0.2**	**0.8 ± 0.1**	**0.7 ± 0.1**	**2.6 ± 0.4**	**2.9 ± 0.5**	**3.1 ± 0.4**	**3.8 ± 0.4**
# assays		8	10	10	10	8	10	8
p value	$KC5^{mdr1\text{-}YF\text{-}1}$			0.68	0.0001	<0.0001	<0.0001	<0.0001
	$KC5^{mdr1\text{-}YY\text{-}1}$				<0.0001	<0.0001	<0.0001	<0.0001
	$KC5^{mdr1\text{-}NF\text{-}1}$					0.41	0.28	0.04
	$KC5^{mdr1\text{-}NF\text{-}2}$						0.41	0.04
	$KC5^{mdr1\text{-}NY\text{-}1}$							0.32
**MFQ IC_{50} (nM)**		**2.9 ± 0.3**	**3.5 ± 0.3**	**2.9 ± 0.2**	**10.1 ± 0.6**	**11.0 ± 0.3**	**12.1 ± 0.9**	**14.1 ± 0.6**
# assays		8	9	10	9	7	9	7
p value	$KC5^{mdr1\text{-}YF\text{-}1}$			0.24	<0.0001	0.0002	<0.0001	0.0002
	$KC5^{mdr1\text{-}YY\text{-}1}$				<0.0001	0.0001	<0.0001	0.0001
	$KC5^{mdr1\text{-}NF\text{-}1}$					0.35	0.05	0.0007
	$KC5^{mdr1\text{-}NF\text{-}2}$						0.25	0.0006
	$KC5^{mdr1\text{-}NY\text{-}1}$							0.14
**DHA IC_{50} (nM)**		**0.53 ± 0.03**	**0.49 ± 0.06**	**0.45 ± 0.04**	**0.73 ± 0.06**	**0.77 ± 0.1**	**0.77 ± 0.04**	**0.77 ± 0.06**
# assays		8	9	9	8	8	9	7
p value	$KC5^{mdr1\text{-}YF\text{-}1}$			0.55	0.0055	0.04	0.0012	0.0052
	$KC5^{mdr1\text{-}YY\text{-}1}$				0.0003	0.0079	<0.0001	0.0007
	$KC5^{mdr1\text{-}NF\text{-}1}$					0.80	0.24	0.69
	$KC5^{mdr1\text{-}NF\text{-}2}$						0.54	0.87
	$KC5^{mdr1\text{-}NY\text{-}1}$							p>0.9999
**PPQ IC_{50} (nM)**		**37.3 ± 3.6**	**34.5 ± 4.0**	**32.0 ± 2.8**	**33.3 ± 3.1**	**30.3 ± 2.5**	**33.7 ± 2.7**	**30.3 ± 1.6**
# assays		8	8	8	8	7	8	7
p value	$KC5^{mdr1\text{-}YF\text{-}1}$			0.96	0.96	0.46	p>0.9999	0.87
	$KC5^{mdr1\text{-}YY\text{-}1}$				0.57	0.69	0.51	0.78
	$KC5^{mdr1\text{-}NF\text{-}1}$					0.46	0.96	0.28
	$KC5^{mdr1\text{-}NF\text{-}2}$						0.34	0.80
	$KC5^{mdr1\text{-}NY\text{-}1}$							0.28

Supplementary Table 4 (page 2 of 2) | Mean±SEM IC$_{50}$ values (nM) of KC5 parental strain and *pfmdr1*-modified recombinant lines.

**CQ IC$_{50}$ (nM)**		**152.0 ± 16.9**	**112.0 ± 6.2**	**110.9 ± 5.9**	**91.8 ± 9.7**	**87.2 ± 7.8**	**78.6 ± 3.4**	**82.5 ± 3.8**
# assays		6	6	6	6	6	6	6
p value	KC5$^{mdr1\text{-YF-1}}$			p>0.9999	0.13	0.04	0.0022	0.0043
	KC5$^{mdr1\text{-YY-1}}$				0.24	0.07	0.0043	0.0087
	KC5$^{mdr1\text{-NF-1}}$					0.82	0.31	0.59
	KC5$^{mdr1\text{-NF-2}}$						0.39	0.59
	KC5$^{mdr1\text{-NY-1}}$							0.48
**md-CQ IC$_{50}$ (nM)**		**716.6 ± 63.4**	**676.8 ± 51.9**	**681.5 ± 43.0**	**579.5 ± 59.1**	**560.8 ± 50.9**	**565.1 ± 49.3**	**574.4 ± 40.0**
# assays		7	9	8	8	8	9	8
p value	KC5$^{mdr1\text{-YF-1}}$			0.96	0.24	0.14	0.19	0.20
	KC5$^{mdr1\text{-YY-1}}$				0.20	0.11	0.06	0.08
	KC5$^{mdr1\text{-NF-1}}$					0.88	0.74	0.80
	KC5$^{mdr1\text{-NF-2}}$						0.96	0.72
	KC5$^{mdr1\text{-NY-1}}$							0.96
**md-ADQ IC$_{50}$ (nM)**		**154.6 ± 7.9**	**128.4 ± 9.6**	**131.0 ± 10.6**	**92.8 ± 3.7**	**89.4 ± 3.9**	**86.7 ± 3.3**	**89.4 ± 4.6**
# assays		8	8	8	8	8	8	8
p value	KC5$^{mdr1\text{-YF-1}}$			0.88	0.0011	0.0011	0.0006	0.0011
	KC5$^{mdr1\text{-YY-1}}$				0.0030	0.0011	0.0006	0.0011
	KC5$^{mdr1\text{-NF-1}}$					0.65	0.20	0.57
	KC5$^{mdr1\text{-NF-2}}$						0.33	0.96
	KC5$^{mdr1\text{-NY-1}}$							0.88
**QN IC$_{50}$ (nM)**		**33.4 ± 2.2**	**34.6 ± 2.2**	**35.3 ± 2.3**	**32.1 ± 1.5**	**32.1 ± 1.5**	**37.9 ± 1.3**	**36.5 ± 1.9**
# assays		7	8	8	7	8	9	8
p value	KC5$^{mdr1\text{-YF-1}}$			0.88	0.46	0.23	0.32	0.33
	KC5$^{mdr1\text{-YY-1}}$				0.28	0.20	0.48	0.80
	KC5$^{mdr1\text{-NF-1}}$					0.78	0.01	0.02
	KC5$^{mdr1\text{-NF-2}}$						0.01	0.13
	KC5$^{mdr1\text{-NY-1}}$							0.74

IC$_{50}$ values (expressed as mean ± SEM). (LMF) lumefantrine, (MFQ) mefloquine, (DHA) dihydroartemisinin, (PPQ) piperaquine, (CQ) chloroquine, (md-CQ) monodesethyl-chloroquine, (md-ADQ) monodesethyl-amodiaquine and (QN) quinine.

Statistical comparisons made using Mann–Whitney U test. Color code:

not significant	p<0.05	p<0.01	p<0.001

Supplementary Table 5 (page 1 of 2) | Mean±SEM IC_{90} values (nM) of NF10 parental strain and *pfmdr1*-modified recombinant lines.

Line	NF10 (YF)	NF10$^{mdr1\text{-YF-1}}$	NF10$^{mdr1\text{-YY-1}}$	NF10$^{mdr1\text{-YY-2}}$	NF10$^{mdr1\text{-NF-1}}$	NF10$^{mdr1\text{-NF-2}}$	NF10$^{mdr1\text{-NY-1}}$	NF10$^{mdr1\text{-NY-2}}$
**LMF IC_{90} (nM)**	**2.8 ± 0.1**	**3.1 ± 0.3**	**3.0 ± 0.3**	**2.9 ± 0.2**	**7.0 ± 0.7**	**6.6 ± 0.7**	**8.8 ± 0.8**	**8.3 ± 1.1**
# assays	7	7	7	7	5	5	6	6
p value								
NF10$^{mdr1\text{-YF-1}}$			0.90	p>0.9999	0.0025	0.0025	0.0012	0.0012
NF10$^{mdr1\text{-YY-1}}$				0.71	0.0025	0.0025	0.0012	0.0012
NF10$^{mdr1\text{-YY-2}}$					0.0025	0.0025	0.0012	0.0012
NF10$^{mdr1\text{-NF-1}}$						0.69	0.18	0.43
NF10$^{mdr1\text{-NF-2}}$							0.13	0.25
NF10$^{mdr1\text{-NY-1}}$								0.70
**MFQ IC_{90} (nM)**	**10.2 ± 0.5**	**10.6 ± 0.4**	**10.2 ± 0.5**	**10.5 ± 0.4**	**26.0 ± 2.6**	**25.5 ± 2.2**	**25.0 ± 1.6**	**25.9 ± 2.4**
# assays	7	7	7	7	7	7	7	7
p value								
NF10$^{mdr1\text{-YF-1}}$			0.54	0.71	0.0006	0.0006	0.0006	0.0006
NF10$^{mdr1\text{-YY-1}}$				0.80	0.0006	0.0006	0.0006	0.0006
NF10$^{mdr1\text{-YY-2}}$					0.0006	0.0006	0.0006	0.0006
NF10$^{mdr1\text{-NF-1}}$						p>0.9999	0.62	0.62
NF10$^{mdr1\text{-NF-2}}$							0.62	0.62
NF10$^{mdr1\text{-NY-1}}$								0.46
**DHA IC_{90} (nM)**	**1.8 ± 0.2**	**1.7 ± 0.2**	**1.8 ± 0.2**	**1.7 ± 0.1**	**2.4 ± 0.2**	**2.3 ± 0.2**	**2.5 ± 0.2**	**2.4 ± 0.2**
# assays	7	7	7	7	7	7	7	7
p value								
NF10$^{mdr1\text{-YF-1}}$			p>0.9999	0.90	0.03	0.07	0.01	0.04
NF10$^{mdr1\text{-YY-1}}$				0.90	0.04	0.07	0.03	0.04
NF10$^{mdr1\text{-YY-2}}$					0.01	0.05	0.0070	0.01
NF10$^{mdr1\text{-NF-1}}$						0.80	0.62	p>0.9999
NF10$^{mdr1\text{-NF-2}}$							0.32	0.80
NF10$^{mdr1\text{-NY-1}}$								p>0.9999
**PPQ IC_{90} (nM)**	**84.5 ± 8.4**	**82.3 ± 7.9**	**76.9 ± 9.8**	**72.2 ± 8.0**	**61.8 ± 2.5**	**61.8 ± 2.7**	**57.4 ± 0.8**	**56.0 ± 1.2**
# assays	6	6	6	6	6	6	6	6
p value								
NF10$^{mdr1\text{-YF-1}}$			0.59	0.48	0.09	0.04	0.0087	0.0087
NF10$^{mdr1\text{-YY-1}}$				p>0.9999	0.82	0.70	0.39	0.13
NF10$^{mdr1\text{-YY-2}}$					0.70	0.39	0.13	0.04
NF10$^{mdr1\text{-NF-1}}$						0.82	0.18	0.03
NF10$^{mdr1\text{-NF-2}}$							0.06	0.06
NF10$^{mdr1\text{-NY-1}}$								0.59

Supplementary Table 5 (page 2 of 2) | Mean±SEM IC_{90} values (nM) of NF10 parental strain and *pfmdr1*-modified recombinant lines.

	**CQ IC_{90} (nM)**	**515.6 ± 33.4**	**514.3 ± 42.3**	**522.1 ± 28.4**	**513.1 ± 36.1**	**408.9 ± 30.2**	**404.3 ± 26.3**	**356.0 ± 27.3**	**361.2 ± 26.2**
	# assays	6	6	6	6	6	6	6	6
p value	NF10$^{mdr1\text{-YF-1}}$			p>0.9999	p>0.9999	0.04	0.03	0.0043	0.0022
	NF10$^{mdr1\text{-YY-1}}$				0.70	0.0022	0.0022	0.0022	0.0022
	NF10$^{mdr1\text{-YY-2}}$					0.0043	0.0043	0.0022	0.0022
	NF10$^{mdr1\text{-NF-1}}$						0.70	0.06	0.0411
	NF10$^{mdr1\text{-NF-2}}$							0.18	0.13
	NF10$^{mdr1\text{-NY-1}}$								0.70
	**md-CQ IC_{90} (nM)**	**1859.4 ± 22.6**	**1866.3 ± 21.0**	**1834.5 ± 44.4**	**1852.6 ± 18.7**	**1726.3 ± 57.9**	**1651.0 ± 61.1**	**1643.9 ± 43.4**	**1514.3 ± 93.1**
	# assays	6	6	6	6	6	6	6	6
p value	NF10$^{mdr1\text{-YF-1}}$			0.82	0.82	0.04	0.0022	0.0022	0.0022
	NF10$^{mdr1\text{-YY-1}}$				0.94	0.24	0.04	0.03	0.0087
	NF10$^{mdr1\text{-YY-2}}$					0.04	0.0022	0.0022	0.0022
	NF10$^{mdr1\text{-NF-1}}$						0.1797	0.18	0.0411
	NF10$^{mdr1\text{-NF-2}}$							0.70	0.18
	NF10$^{mdr1\text{-NY-1}}$								0.31
	**md-ADQ IC_{90} (nM)**	**115.7 ± 1.3**	**116.4 ± 1.0**	**115.7 ± 1.3**	**116.5 ± 1.0**	**72.8 ± 7.7**	**65.8 ± 5.7**	**59.4 ± 0.5**	**58.7 ± 0.3**
	# assays	6	6	6	6	6	6	6	6
p value	NF10$^{mdr1\text{-YF-1}}$			0.82	0.94	0.0022	0.0022	0.0022	0.0022
	NF10$^{mdr1\text{-YY-1}}$				0.82	0.0022	0.0022	0.0022	0.0022
	NF10$^{mdr1\text{-YY-2}}$					0.0022	0.0022	0.0022	0.0022
	NF10$^{mdr1\text{-NF-1}}$						0.39	0.06	0.0087
	NF10$^{mdr1\text{-NF-2}}$							0.24	0.03
	NF10$^{mdr1\text{-NY-1}}$								0.39
	**QN IC_{90} (nM)**	**310.6 ± 3.8**	**312.7 ± 11.0**	**315.0 ± 10.2**	**297.6 ± 10.6**	**202.8 ± 16.6**	**216.3 ± 29.7**	**220.7 ± 15.4**	**219.9 ± 22.0**
	# assays	5	5	5	5	5	5	5	5
p value	NF10$^{mdr1\text{-YF-1}}$			p>0.9999	0.69	0.0079	0.10	0.0079	0.02
	NF10$^{mdr1\text{-YY-1}}$				0.42	0.0079	0.10	0.0079	0.0079
	NF10$^{mdr1\text{-YY-2}}$					0.02	0.15	0.02	0.03
	NF10$^{mdr1\text{-NF-1}}$						0.69	0.31	0.42
	NF10$^{mdr1\text{-NF-2}}$							0.31	0.84
	NF10$^{mdr1\text{-NY-1}}$								0.55

IC_{90} values (expressed as mean ± SEM). (LMF) lumefantrine, (MFQ) mefloquine, (DHA) dihydroartemisinin, (PPQ) piperaquine, (CQ) chloroquine, (md-CQ) monodesethyl-chloroquine, (md-ADQ) monodesethyl-amodiaquine and (QN) quinine.

Statistical comparisons made using Mann–Whitney U test.

Color code:	not significant	p<0.05	p<0.01	p<0.001

Supplementary Table 6 (page 1 of 2) | Mean±SEM IC_{90} values (nM) of KC5 parental strain and *pfmdr1*-modified recombinant lines.

Line	KC5 (YF)	KC5$^{mdr1\text{-YF-1}}$	KC5$^{mdr1\text{-YY-1}}$	KC5$^{mdr1\text{-NF-1}}$	KC5$^{mdr1\text{-NF-2}}$	KC5$^{mdr1\text{-NY-1}}$	KC5$^{mdr1\text{-NY-2}}$
**LMF IC_{90} (nM)**	**3.0 ± 0.5**	**2.9 ± 0.2**	**2.8 ± 0.2**	**11.1 ± 1.4**	**10.6 ± 0.8**	**10.3 ± 0.6**	**12.1 ± 0.6**
# assays	8	10	10	10	8	10	8
p value							
KC5$^{mdr1\text{-YF-1}}$			0.74	0.0001	<0.0001	<0.0001	<0.0001
KC5$^{mdr1\text{-YY-1}}$				<0.0001	<0.0001	<0.0001	<0.0001
KC5$^{mdr1\text{-NF-1}}$					0.83	0.74	0.46
KC5$^{mdr1\text{-NF-2}}$						0.63	0.23
KC5$^{mdr1\text{-NY-1}}$							0.02
**MFQ IC_{90} (nM)**	**9.2 ± 1.1**	**9.4 ± 0.5**	**9.0 ± 0.4**	**27.1 ± 2.2**	**25.1 ± 1.2**	**26.0 ± 1.2**	**28.8 ± 1.8**
# assays	8	9	10	9	7	9	7
p value							
KC5$^{mdr1\text{-YF-1}}$			0.66	<0.0001	0.0002	<0.0001	0.0002
KC5$^{mdr1\text{-YY-1}}$				<0.0001	0.0001	<0.0001	0.0001
KC5$^{mdr1\text{-NF-1}}$					0.84	0.86	0.35
KC5$^{mdr1\text{-NF-2}}$						0.54	0.10
KC5$^{mdr1\text{-NY-1}}$							0.30
**DHA IC_{90} (nM)**	**1.3 ± 0.1**	**1.6 ± 0.2**	**1.4 ± 0.1**	**1.7 ± 0.2**	**2.0 ± 0.2**	**2.3 ± 0.2**	**2.3 ± 0.2**
# assays	8	9	9	8	8	9	7
p value							
KC5$^{mdr1\text{-YF-1}}$			0.54	0.54	0.07	0.0056	0.0052
KC5$^{mdr1\text{-YY-1}}$				0.16	0.01	0.0011	0.0010
KC5$^{mdr1\text{-NF-1}}$					0.44	0.01	0.05
KC5$^{mdr1\text{-NF-2}}$						0.11	0.23
KC5$^{mdr1\text{-NY-1}}$							p>0.9999
**PPQ IC_{90} (nM)**	**56.3 ± 5.0**	**68.6 ± 9.0**	**62.2 ± 5.5**	**51.2 ± 4.5**	**52.3 ± 3.8**	**52.7 ± 3.9**	**55.2 ± 1.5**
# assays	8	8	8	8	7	8	7
p value							
KC5$^{mdr1\text{-YF-1}}$			0.96	0.38	0.07	0.28	0.19
KC5$^{mdr1\text{-YY-1}}$				0.13	0.07	0.04	0.19
KC5$^{mdr1\text{-NF-1}}$					0.54	p>0.9999	0.96
KC5$^{mdr1\text{-NF-2}}$						0.19	p>0.9999
KC5$^{mdr1\text{-NY-1}}$							0.46

Supplementary Table 6 (page 2 of 2) | Mean±SEM IC_{90} values (nM) of KC5 parental strain and *pfmdr1*-modified recombinant lines.

**CQ IC_{90} (nM)**	**368.2 ± 45.2**	**258.3 ± 18.2**	**263.6 ± 26.1**	**300.1 ± 45.1**	**223.5 ± 22.1**	**194.8 ± 16.4**	**218.5 ± 22.5**
# assays	6	6	6	6	6	6	6
p value KC5$^{mdr1\text{-YF-1}}$			0.82	0.59	0.70	0.02	0.09
p value KC5$^{mdr1\text{-YY-1}}$				0.49	0.82	0.03	0.18
p value KC5$^{mdr1\text{-NF-1}}$					0.13	0.09	0.24
p value KC5$^{mdr1\text{-NF-2}}$						0.24	0.70
p value KC5$^{mdr1\text{-NY-1}}$							0.49
**md-CQ IC_{90} (nM)**	**1598.3 ± 102.3**	**1476.4 ± 98.9**	**1513.0 ± 109.7**	**1586.7 ± 139.9**	**1449.7 ± 113.0**	**1277.7 ± 125.9**	**1427.7 ± 106.2**
# assays	7	9	8	8	8	9	8
p value KC5$^{mdr1\text{-YF-1}}$			0.89	0.48	0.82	0.30	0.82
p value KC5$^{mdr1\text{-YY-1}}$				0.44	0.72	0.14	0.44
p value KC5$^{mdr1\text{-NF-1}}$					0.38	0.17	0.33
p value KC5$^{mdr1\text{-NF-2}}$						0.37	0.96
p value KC5$^{mdr1\text{-NY-1}}$							0.37
**md-ADQ IC_{90} (nM)**	**235.0 ± 2.3**	**210.4 ± 9.0**	**212.9 ± 9.3**	**163.9 ± 12.3**	**140.4 ± 11.6**	**138.2 ± 11.6**	**138.0 ± 13.0**
# assays	8	8	8	8	8	8	8
p value KC5$^{mdr1\text{-YF-1}}$			p>0.9999	0.01	0.0019	0.0019	0.0019
p value KC5$^{mdr1\text{-YY-1}}$				0.01	0.0011	0.0019	0.0019
p value KC5$^{mdr1\text{-NF-1}}$					0.16	0.28	0.19
p value KC5$^{mdr1\text{-NF-2}}$						0.65	0.65
p value KC5$^{mdr1\text{-NY-1}}$							0.80
**QN IC_{90} (nM)**	**194.9 ± 34.8**	**131.6 ± 15.3**	**147.4 ± 15.9**	**267.3 ± 69.1**	**192.2 ± 44.8**	**153.2 ± 33.9**	**210.5 ± 45.9**
# assays	7	8	8	7	8	9	8
p value KC5$^{mdr1\text{-YF-1}}$			0.65	0.05	0.80	0.54	p>0.9999
p value KC5$^{mdr1\text{-YY-1}}$				0.15	p>0.9999	0.24	0.96
p value KC5$^{mdr1\text{-NF-1}}$					0.40	0.17	0.69
p value KC5$^{mdr1\text{-NF-2}}$						0.48	0.96
p value KC5$^{mdr1\text{-NY-1}}$							0.48

IC_{90} values (expressed as mean ± SEM). (LMF) lumefantrine, (MFQ) mefloquine, (DHA) dihydroartemisinin, (PPQ) piperaquine, (CQ) chloroquine, (md-CQ) monodesethyl-chloroquine, (md-ADQ) monodesethyl-amodiaquine and (QN) quinine.

Statistical comparisons made using Mann–Whitney U test. Color code:

not significant	p<0.05	p<0.01	p<0.001

Supplementary Table 7 | Mean±SEM IC_{50} values of K1 parental strain and PfMDR1 F1226Y recombinant lines.

Line		K1 (SNFD)	K1$^{mdr1\text{-SNFD}}$	K1$^{mdr1\text{-SNYD-1}}$	K1$^{mdr1\text{-SNYD-2}}$
**LMF IC_{50} (nM)**		**2.6 ± 1.1**	**1.8 ± 0.6**	**1.5 ± 0.6**	**2.0 ± 0.6**
# assays		3	3	3	3
p value	K1 (SNFD)		0.70	0.40	0.90
	K1$^{mdr1\text{-SNFD}}$			0.70	0.90
	K1$^{mdr1\text{-SNYD-1}}$				0.40
**MFQ IC_{50} (nM)**		**10.1 ± 1.4**	**9.4 ± 0.9**	**7.3 ± 1.2**	**8.8 ± 0.7**
# assays		4	4	4	4
p value	K1 (SNFD)		p>0.9999	0.20	0.66
	K1$^{mdr1\text{-SNFD}}$			0.20	0.66
	K1$^{mdr1\text{-SNYD-1}}$				0.20
**ART IC_{50} (nM)**		**2.7 ± 0.3**	**2.4 ± 0.4**	**2.1 ± 0.2**	**2.3 ± 0.3**
# assays		4	4	4	4
p value	K1 (SNFD)		0.66	0.20	0.34
	K1$^{mdr1\text{-SNFD}}$			p>0.9999	p>0.9999
	K1$^{mdr1\text{-SNYD-1}}$				0.66
**AS IC_{50} (nM)**		**1.9 ± 0.3**	**1.7 ± 0.2**	**1.4 ± 0.2**	**1.7 ± 0.2**
# assays		4	4	4	4
p value	K1 (SNFD)		0.83	0.20	0.66
	K1$^{mdr1\text{-SNFD}}$			0.49	0.83
	K1$^{mdr1\text{-SNYD-1}}$				0.34
**PPQ IC_{50} (nM)**		**16.1 ± 1.7**	**18.7 ± 1.9**	**15.8 ± 1.6**	**17.0 ± 1.7**
# assays		4	4	4	4
p value	K1 (SNFD)		0.49	p>0.9999	0.83
	K1$^{mdr1\text{-SNFD}}$			0.20	0.66
	K1$^{mdr1\text{-SNYD-1}}$				0.66
**CQ IC_{50} (nM)**		**278.3 ± 23.7**	**312.8 ± 31.2**	**269.6 ± 29.6**	**292.1 ± 25.4**
# assays		4	4	4	4
p value	K1 (SNFD)		0.66	0.66	0.49
	K1$^{mdr1\text{-SNFD}}$			0.49	0.66
	K1$^{mdr1\text{-SNYD-1}}$				0.34
**md-ADQ IC_{50} (nM)**		**70.2 ± 4.5**	**81.6 ± 7.1**	**79.4 ± 6.9**	**83.8 ± 5.6**
# assays		4	4	4	4
p value	K1 (SNFD)		0.34	0.34	0.20
	K1$^{mdr1\text{-SNFD}}$			0.83	p>0.9999
	K1$^{mdr1\text{-SNYD-1}}$				0.49
**QN IC_{50} (nM)**		**125.5 ± 21.4**	**115.8 ± 26.8**	**130.6 ± 25.0**	**124.4 ± 25.1**
# assays		4	4	4	4
p value	K1 (SNFD)		p>0.9999	p>0.9999	p>0.9999
	K1$^{mdr1\text{-SNFD}}$			0.66	0.66
	K1$^{mdr1\text{-SNYD-1}}$				0.66

IC_{50} values (expressed as mean ± SEM). (LMF) lumefantrine; (MFQ) mefloquine; (ART) artemisinin; (AS) artesunate; (PPQ) piperaquine; (CQ) chloroquine; (md-ADQ) monodesethyl-amodiaquine; (QN) quinine. (SNFD) K1 PfMDR1 haplotype at positions 1034, 1042, 1226, 1246 respectively. Statistical comparisons were made using Mann–Whitney *U* test.

Supplementary Table 8 | List of oligonucleotides used in this study.

Primer Name	Description		Primer sequence (5'-3')
p1	529 bp upstream of *pfmdr1* start codon; tailed with BstAPI restriction site (underlined)	Fwd	gtAgcataTggtgCATGGATATAGAAAGAAAAATGGAAG
p2	1775 bp downstream of *pfmdr1* start codon; tailed with AatII restriction site (underlined)	Rev	gTTGacgTcTGTACTAAATACTCAGATTTATTATCTAAAG
p3	627 bp upstream of *pfmdr1* start codon	Fwd	CATTTTTACTGATTCCTTTAAAAGGC
p4	709 bp downstream of *pfmdr1* start codon	Rev	CCTCTTCTATAATGGACATGGTATTG
p5	3217 bp downstream of *pfmdr1* start codon	Fwd	ACTGGTAGTTATGCTGGAAAATTA
p6	Anneals at the start of the *P. yoelii* mdr1 3' UTR	Rev	TTATCTGGTTATGTATCTGCTCA
p7	1758 bp downstream of *pfmdr1* start codon	Fwd	TGATGAAGCTACATCTTCTTTAG
p8	Anneals downstream of *pfmdr1*	Rev	ACGGACAAGAGTTGATACTGTTCAT
p9	SDM primer for engineering 3 silent mutations (small letters) under the ZFN binding site	Fwd	GTAACtTgAGTATCAAAGAgGAGGTTGAAAAAGAGTTGAAC
p10	SDM primer for engineering 3 silent mutations (small letters) under the ZFN binding site	Rev	CTCcTCTTTGATACTcAaGTTACCATCTTTTTTCTCTTTCTG
p11	SDM primer for engineering Y86 (small letter) in *pfmdr1*	Fwd	GTTTGGTGTAATATTAAAGtACATGTATTTAGGTGATGATATTAATCC
p12	SDM primer for engineering Y86 (small letter) in *pfmdr1*	Rev	GGATTAATATCATCACCTAAATACATGTaCTTTAATATTACACCAAAC
p13	SDM primer for engineering F184 (small letter) in *pfmdr1*	Fwd	CCAGTTCCTTTTTAGGTTTATaTATTTGGTCATTAATAAAAAATG
p14	SDM primer for engineering F184 (small letter) in *pfmdr1*	Rev	CATTTTTTATTAATGACCAAATAtATAAACCTAAAAAGGAACTGG
p15	SDM primer for engineering F1226Y (small letter) in *pfmdr1*	Fwd	GGATCTGCAGAAGATTATACTGTATaTAATAATAATGGAGAAATATTATTAG
p16	SDM primer for engineering F1226Y (small letter) in *pfmdr1*	Rev	CTAATAATATTTCTCCATTATTATTAtATACAGTATAATCTTCTGCAGATCC
p17	Anneals in *pfmdr1* 5' UTR	Fwd	CAAATAAATAAATACGTATGTGAGC
p18	2619 bp downstream of *pfmdr1* start codon	Rev	TGATCAAAGAAACTCATTTCTTGA

SDM: site directed mutagenesis; ZFN: zinc finger nucleases
